# Risk of Rabies and Implications for Postexposure Prophylaxis Administration in the US

**DOI:** 10.1001/jamanetworkopen.2023.17121

**Published:** 2023-06-09

**Authors:** Kelly Charniga, Yoshinori Nakazawa, Jen Brown, Seonghye Jeon, Ryan M. Wallace

**Affiliations:** 1 Division of High-Consequence Pathogens and Pathology, National Center for Emerging and Zoonotic Infectious Diseases, US Centers for Disease Control and Prevention, Atlanta, Georgia; 2 Indiana Department of Health, Indianapolis; 3 Division of Preparedness and Emerging Infections, National Center for Emerging and Zoonotic Infectious Diseases, US Centers for Disease Control and Prevention, Atlanta, Georgia

## Abstract

**Importance:**

In the US, rabies postexposure prophylaxis (PEP) is often administered without a comprehensive and regionally appropriate rabies risk assessment. For low-risk exposures, this can result in patients incurring out-of-pocket expenses or experiencing adverse effects of PEP unnecessarily.

**Objective:**

To use a model to estimate (1) the probability that an animal would test positive for rabies virus (RABV) given that a person was exposed, and (2) the probability that a person would die from rabies given that they were exposed to a suspect rabid animal and did not receive PEP, and to propose a risk threshold for recommending PEP according to model estimates and a survey.

**Design, Setting, and Participants:**

In this decision analytical modeling study, positivity rates were calculated using more than 900 000 animal samples tested for RABV between 2011 and 2020. Other parameters were estimated from a subset of the surveillance data and the literature. Probabilities were estimated using Bayes’ rule. A survey was administered among a convenience sample of state public health officials in all US states (excluding Hawaii) plus Washington, DC and Puerto Rico to determine a risk threshold for PEP recommendation. Respondents were asked whether they would recommend PEP given 24 standardized exposure scenarios while accounting for local rabies epidemiology.

**Main Outcomes and Measures:**

A quantitative and regionally appropriate approach for helping health care practitioners and public health professionals determine whether to recommend and/or administer rabies PEP.

**Results:**

A total of 1728 unique observations were obtained from the model for the probability that an animal would test positive for RABV given that a person was exposed, and 41 472 for ) the probability that a person would die from rabies given that they were exposed to a suspect rabid animal and did not receive PEP. The median probability that an animal would test positive for RABV given that a person was exposed ranged from 3 × 10^−7^ to 0.97, while the probability that a person would die from rabies given that they were exposed to a suspect rabid animal and did not receive PEP ranged from 1 × 10^−10^ to 0.55. Fifty public health officials out of a target sample size of 102 responded to the survey. Using logistic regression, a risk threshold was estimated for PEP recommendation of 0.0004; PEP may not be recommended for exposures with probabilities below this threshold.

**Conclusions and Relevance:**

In this modeling study of rabies in the US, the risk of death|exposure was quantified and a risk threshold was estimated. These results could be used to inform the decision-making process as to the appropriateness of recommending rabies PEP.

## Key Points

**Question:**

Could a quantitative rabies risk assessment model help inform public health professionals’ recommendations for rabies postexposure prophylaxis (PEP)?

**Findings:**

In this decision analytical modeling study, the conditional probability that an animal was rabid, given that a person was exposed, was estimated while accounting for regional differences in rabies epidemiology. A survey of 50 state public health veterinarians was used to estimate a risk threshold of 0.0004 for PEP administration.

**Meaning:**

These estimates may help health care practitioners and public health professionals conduct objective and regionally appropriate rabies risk assessments, particularly when an animal is not available for testing or observation.

## Teaser

This decision analytical model evaluates the risk of rabies and implications for postexposure prophylaxis administration in the US.

## Introduction

Rabies is one of the world’s deadliest diseases, with a fatality rate near 100%.^1^ It is endemic in most countries, including the US, where main reservoirs are wildlife species.^2^ Rabies virus (RABV) is transmitted to humans via direct contact with the saliva, brain, or nervous system tissue of an infected animal. The virus enters the body through broken skin or mucous membranes.^3^ Rabies can be prevented by avoiding animal bites and through timely administration of postexposure prophylaxis (PEP) following exposure to a suspect rabid animal.

Most physicians in the US will never treat a human rabies case.^1^ However, animal bites from pets and wildlife are a common cause of injury, resulting in hundreds of thousands of primary care and emergency department visits each year and requiring physicians to routinely make decisions about whether to administer PEP.^4,5^ Rabies PEP for immune competent and previously unvaccinated persons consists of 4 doses of rabies vaccine and 1 dose of immune globulin administered over a 2-week period.^6^ PEP prevents the disease in nearly every case if given before the onset of symptoms. An estimated 55 000 (range 45 453-66 000) people in the US received treatment for potential RABV exposures each year from 2017 to 2018, at an estimated cost of more than $3800 per person treated.^1^

Rabies PEP guidelines are published by the Advisory Committee on Immunization Practices.^6,7^ In addition, most public health jurisdictions have their own guidelines for rabies risk assessments and testing suspect rabid animals, which typically consist of a static flowchart posted on the agency website.^8,9,10,11,12^ Both retrospective and prospective studies in the US have found that guidelines for when to administer PEP are often not followed.^13,14,15^ Common reasons for inappropriate PEP administration include (1) the animal tested negative for RABV,^15^ (2) the patient had no known animal contact,^15^ and (3) the bite was low risk for rabies.^15^ In this study, we focus on issues related to the third reason.

Providing PEP when it is not warranted is costly for patients, health systems, and public health departments.^1^ Additionally, rabies vaccine has been associated with rare adverse events, including injection site reactions, systemic hypersensitivity reactions, and neurological complications.^7^ There have also been supply chain disruptions of rabies biologics in the US, particularly in the mid-2000s, due to unexpected decreases in supply.^16^ For these reasons, rabies PEP should be used judiciously and only after a risk assessment has been conducted by a health care professional.

Risk factors for rabies in animals include species, geographic location, health status, and vaccination status. Each of the 5 terrestrial rabies reservoir species (eg, skunk, fox, raccoon, mongoose, and arctic fox) in the US is associated with a particular RABV variant and distinct geographic boundary.^2^ An animal that appears ill or is acting strangely is more likely to be rabid than if it were healthy and behaving normally.^17,18^ Clinical signs of rabies in animals can include hypersalivation, paralysis, lethargy, aggression, abnormal vocalization, and diurnal activity of nocturnal species; however, RABV can be shed several days before clinical onset, and clinical signs alone are not reliable for making risk assessment determinations.^19^ Rabies vaccines reduce the risk of rabies and are licensed for several domestic animals, including dogs, cats, ferrets, horses, sheep, and cattle, as well as coyotes and raccoons.^20^ Other livestock and zoo animals may also receive rabies vaccine.^20^

The circumstances surrounding a potential RABV exposure are also important in assessing rabies risk. Unprovoked exposures are those for which aggression is not explained by normal animal behavior, whereas provoked exposures may involve feeding an animal, pulling its tail, trespassing on its territory, threatening its offspring, and so forth.^21^ Unprovoked animal attacks on humans are rare and are more likely to involve rabid animals than provoked attacks.^7,21^

There are few examples of national algorithms for rabies risk assessments for exposures that occur in the US. While these tools do not replace expert rabies knowledge and experience, they help to prevent human rabies deaths and avoid PEP administration when it is not needed. The Rabies Algorithm is a publicly available flowchart developed by the Wisconsin Department of Health Services and is based heavily on 2008 ACIP guidelines.^22^ It addresses the most common and straightforward rabies exposure scenarios. It includes 6 animal categories and does not account for geographic heterogeneity in rabies risk. The tool has been a helpful training resource, but its lack of diverse and regionally-specific exposure scenarios has limited its use.

The aim of this analysis is to develop a probabilistic risk assessment model and tool using data from the US Centers for Disease Control and Prevention (CDC) National Rabies Surveillance System (NRSS) and the literature. Risk estimates could be used to assist healthcare providers and health departments in making decisions regarding appropriate rabies PEP administration, particularly when an animal is not available for RABV testing or observation.

## Methods

The collection and analysis of rabies surveillance data was reviewed by CDC and was conducted consistent with applicable federal law and CDC policy (45 C.F.R. part 46.102(l)(2), 21 C.F.R. part 56; 42 U.S.C. §241(d); 5 U.S.C. §552a; 44 U.S.C. §3501 et seq.). Analysis of the survey data collected by the National Association of State Public Health Veterinarians (NASPHV) received a nonresearch determination by a CDC human participants advisor. This report follows the Consolidated Health Economic Evaluation Reporting Standards (CHEERS) reporting guideline for economic analyses, decision-analytic models, or simulated modeling studies.^29^

**Figure 1.**
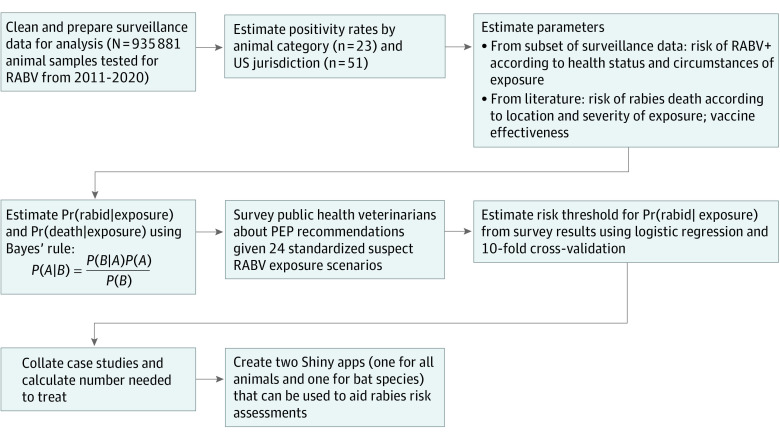
Analysis Workflow for Rabies Risk Assessment Tools PEP indicates postexposure prophylaxis; Pr(death|exposure), the probability that an animal would test positive for rabies virus given that a person was exposed; RABV, rabies virus.

Information on 935 881 animal samples reported to the NRSS from 51 public health jurisdictions between 2011 and 2020 were available for this analysis (Figure 1 and eAppendix 1, eMethods, and eFigure 1 in Supplement 1). A logistic regression approach to estimate the risk of rabies was not feasible because only 4 predictor variables were routinely collected and reported to the NRSS (jurisdiction, animal type, test result, and year), and the proportion of positive samples was low (0.06). Instead, we used Bayes’ rule to estimate (1) the probability that an animal would test positive for RABV given that a person was exposed, Pr(rabid|exposure) and (2) the probability that a person would die from rabies given that they were exposed to a suspect rabid animal and did not receive PEP, Pr(death|exposure).

### Positivity Rates

We first classified animal samples into 23 mutually-exclusive risk groups for rabies according to existing guidance,^2,22,23^ taxonomy,^24^ diet (eg, herbivore vs omnivore),^25^ and geographic origin (native vs nonnative wild) (eTable 1 in Supplement 1).^25^ We calculated positivity rates for each animal category as the number of samples that tested positive divided by the total number of samples with a positive or negative test result (eFigure 2 in Supplement 1). We used jurisdiction-specific positivity rates for some animal categories and pooled estimates for others, primarily according to data availability and epidemiologic relevance (eFigure 3, eFigure 4, eFigure 5, eFigure 6, and eTable 1 in Supplement 1).

### Parameter Estimates

Animal health status and exposure circumstances are not reported to the NRSS by all jurisdictions and do not undergo additional data validation processes. We reviewed 357 datafiles to determine if these variables were present in the jurisdiction’s original submission for 2014 to 2020 (in 2014, jurisdictions routinely began submitting linelist data rather than aggregate county counts). We assumed vaccine effectiveness of 95% in animals^26,27^ and that the vaccination status of coyotes and raccoons would be unknown. We searched Google Scholar, PubMed, and the gray literature for estimates of the probability of dying from rabies by anatomical location of the exposure and severity of viral inoculation. We used a snowball search strategy because most references were historical (eMethods in Supplement 1).

### Model

To estimate Pr(rabid|exposure), we sampled from the binomial distribution of the positivity rates 1 million times for each animal category and jurisdiction (for the jurisdiction-specific animals). Then we updated each sampled probability for animal health status using Bayes’ rule.^28^ Next we updated the probabilities again for exposure circumstances. Finally, we multiplied the result by (1 − vaccine effectiveness) for domestic animals that were up to date with their rabies vaccines. We obtained 95% credible intervals for our estimates by taking the 0.025 and 0.975 quantiles of the resulting distributions. To estimate the Pr(death|exposure), we sampled 1 million times from the distribution of the probability of a person dying from rabies after being exposed to a rabid animal according to anatomical location and severity of exposure and multiplied the result by Pr(rabid|exposure).

### Statistical Analysis

We collaborated with NASPHV to survey state and local public health officials who routinely conduct qualitative rabies risk assessments for the purpose of recommending PEP (eFigure 7 and eAppendix 2 in Supplement 1). Respondents were asked whether they would recommend PEP given 24 standardized suspect RABV exposure scenarios, considering the epidemiology of rabies in their jurisdiction. We used the survey results to identify a universal “risk threshold” for Pr(rabid|exposure) under which PEP may not be recommended and above which PEP would be recommended.

To estimate the risk threshold, we first matched the survey scenarios with the Pr(rabid|exposure) from our analysis. We calculated Spearman rank correlation coefficient between the probabilities and the ordinal survey responses. We fitted a logistic regression model using the natural log of the median Pr(rabid|exposure) as the predictor (the transformation helped meet model assumptions). The outcome was whether PEP was recommended (Yes = “strongly agree” or “agree”; No = “strongly disagree” or “disagree”). The risk threshold was the Pr(rabid|exposure) associated with the decision boundary *P* = .50 of the logistic regression curve (eMethods, eFigure 8, eTable 2 in Supplement 1). All analyses were performed in R version 4.1.1 (R Project for Statistical Computing).

#### Case Studies and the Number Needed to Treat

We examined case studies and calculated the number needed to treat (NNT). The NNT is the number of patients that need to be treated with PEP to prevent 1 additional bad outcome (ie, death due to rabies) (eMethods in Supplement 1).

#### R Shiny Applications

Shiny is a package from RStudio that can be used to create interactive web pages with R. We created 2 Shiny applications using our estimates that could be used by public health officials to aid in rabies risk assessments, one that applies to all animal exposures in the US and the other for bat species (eMethods in Supplement 1).

## Results

### Modeled Probabilities

We obtained 1728 unique observations in the resulting data set for Pr(rabid|exposure) and 41 472 for Pr(death|exposure) (there were 5496 and 153 888 total estimates respectively after repeating pooled estimates for each jurisdiction and including estimates in which the exposure involved a lick to intact skin) (eMethods in Supplement 1). The median Pr(rabid|exposure) ranged from 3 × 10^−7^ to 0.97, while Pr(death|exposure) ranged from 1 × 10^−10^ to 0.55 (Table; eFigure 9 in Supplement 1). The riskiest exposures were unprovoked and involved raccoons, skunks, and foxes that were ill or acting strangely, but the level of risk varied geographically. Mongooses in Puerto Rico also posed a sizable risk. The least risky exposures were provoked and involved cats, dogs, and pocket pets that were vaccinated and apparently healthy at the time of the exposure. Final parameter estimates can be found in eResults, eFigure 10, eTable 3, and eTable 4 in Supplement 1.

**Table.** Summary Statistics of the Median Probability That an Animal Would Test Positive For Rabies Virus (1728 Observations) and the Probability That a Person Would Die From Rabies (41 472 Observations)^a^

Statistic	Pr(rabid|exposure)	Pr(death|exposure)
Min	3 × 10^−7^	1 × 10^−10^
1st quartile	0.0004	2 × 10^−6^
Median	0.0064	0.00005
Mean	0.098	0.0093
3rd quartile	0.076	0.0007
Max	0.97	0.55

^a^ Pooled estimates were only included once (rather than repeated for each jurisdiction) and estimates from lick to intact skin (0 risk of rabies) were removed (see eMethods in Supplement 1 for details). Data sources for the estimates included the national rabies surveillance system (2011-2020) and the literature.

### Risk Threshold

**Figure 2.**
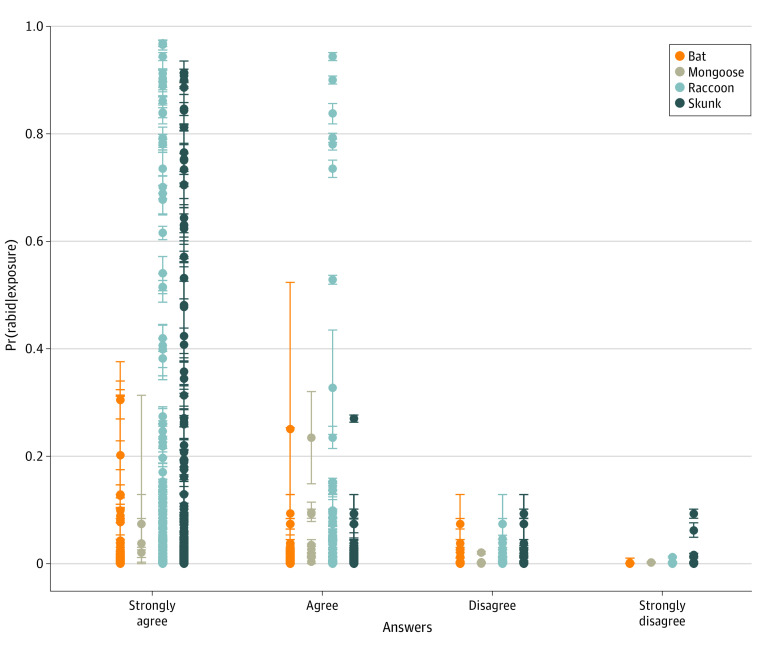
Quantitative Risk Assessment Survey Results for Whether Rabies Postexposure Prophylaxis (PEP) Would Be Recommended Given 24 Scenarios Describing Possible Rabies Virus Exposures The survey was administered to state and local public health officials in the US with experience making professional recommendations regarding rabies PEP. Spearman rank correlation was −0.49 (*P* < .001). Median and 95% credible intervals (error bars) corresponding to each scenario are shown. Colors indicate the main rabies virus reservoir for each jurisdiction. Some scenarios in the “strongly disagree” category were outliers (eTable 5 in Supplement 1).

Survey results are shown in Figure 2. Survey responses were moderately correlated with Pr(rabid|exposure) (Spearman rank correlation, −0.49; *P* < .001). Additional survey results as well as sensitivity analyses can be found in eResults, eFigure 11, eFigure 12, and eTable 5 in Supplement 1.

**Figure 3.**
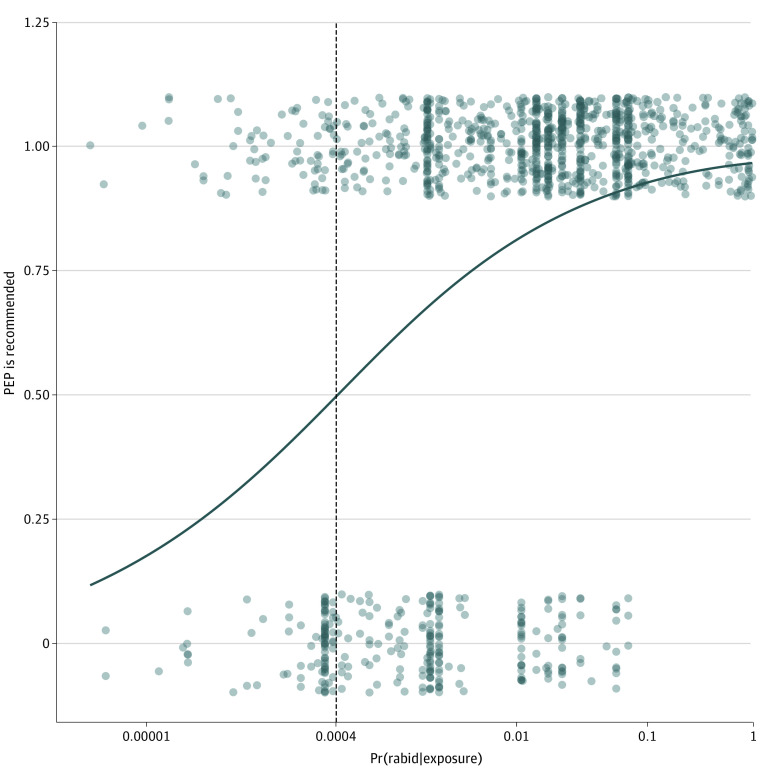
Logistic Regression Predicting Whether Rabies Postexposure Prophylaxis (PEP) Would Be Recommended The vertical dotted line indicates the risk threshold (0.0004). Although the outcome is exclusively binary (PEP is not recommended = 0, or PEP is recommended = 1), we jittered the points and added transparency to avoid overlap and improve readability. Pr(death|exposure) indicates the probability that a person would die from rabies given that they were exposed to a suspect rabid animal and did not receive PEP.

We obtained a risk threshold of 0.0004 from the logistic regression model fitted to the survey data (Figure 3). By applying the risk threshold to all combinations of the 5 risk assessment variables (eg, jurisdiction, species, health status, circumstances, and vaccination status), we found 599 scenarios (11%) were strictly below the threshold, 1274 scenarios (23%) overlapped with the threshold, and 3623 scenarios (66%) were strictly above the threshold, considering the uncertainty in the Pr(rabid|exposure). Note that not all combinations of the risk assessment variables we examined are equally likely to occur (eg, it is far less common to be exposed to a fox, which is high risk in nearly all scenarios compared with a dog, which is low risk in nearly all scenarios). We checked the assumptions of the logistic regression model and investigated potential outliers (eFigure 13 in Supplement 1). Examples of how the estimates from this analysis have been used can be found in eAppendix 3 in Supplement 1.

### Number Needed to Treat

**Figure 4.**
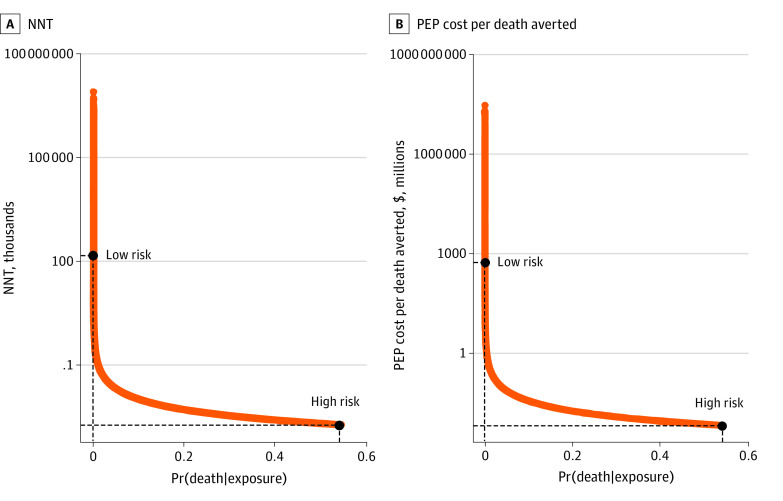
Number Needed to Treat (NNT) to Prevent 1 Rabies Death and Postexposure Prophylaxis (PEP) Cost per Death Averted by the Probability of Death Given Human Exposure to a Suspect Rabid Animal, Pr(death|exposure) Examples of low- and high-risk exposures are shown by the dashed lines. The low-risk exposure corresponds to a single bite to the arm from a vaccinated cat in Michigan that was ill and provoked, while the high-risk exposure corresponds to multiple bites to the head by a skunk in Georgia that was unprovoked and ill or acting strangely.

The NNT for all estimates of Pr(death|exposure) and the PEP cost per death averted is shown in Figure 4. For high estimates of Pr(death|exposure), few people need to be treated to prevent 1 death. For example, the NNT for multiple bites to the head by a skunk in Georgia that was unprovoked and ill or acting strangely is 1 / 0.54 = 1.85. This value is lower than the estimated NNT of rapid defibrillation for cardiac arrest, a common emergency medical intervention for which 2.5 people need to be treated to prevent 1 death.^30^ In contrast, many people need to be treated to prevent 1 death from a low-risk exposure: a single bite to the arm from a vaccinated cat in Michigan that was ill and provoked has an NNT of 1/0.000007 = 143 000. Public health interventions with comparably high NNTs are generally not available due to lack of data (large, randomized controlled trials are needed).

## Discussion

The tools from this decision analytical model improve upon an existing national rabies risk assessment tool^31^ by adding animal categories, accounting for geographic differences in rabies epidemiology, and providing quantitative estimates of risk with uncertainty. The estimates from our analysis could aid health care practitioners and public health officials in risk communication efforts across the spectrum of potential RABV exposure scenarios. For example, they could bolster the argument for PEP among people with high-risk exposures who are hesitant about treatment due to cost,^1^ fear of needles^32^ or vaccines,^33^ lack of paid sick time,^34^ and so forth. Additionally, the estimates could persuade individuals with very low-risk exposures who insist on PEP that treatment may not be necessary.^35^ Approximately one-fourth of the exposures examined in this study overlapped with the estimated risk threshold. PEP should be carefully considered for these situations and may be recommended out of an abundance of caution.

Our modeling framework could be generalized to other countries with robust passive surveillance systems in place.^36^ However, the tools should not be used in other countries due to differences in rabies epidemiology. Unlike the US, most rabies deaths globally are caused by domestic dogs.^37^ Our tools would not be appropriate for countries with mostly rabid dog cases because the model relies on the animal species involved in exposures.

The most common mammals involved in bite events in the US are dogs, cats, and small rodents.^4^ These species, when healthy and provoked into biting, represent some of the lowest risk exposures evaluated in this model (of the scenarios strictly below the risk threshold, more than half were exposures to dogs or cats). PEP is not reportable in all states^38^ and is not considered nationally notifiable to CDC.^39^ This means there are no data to understand the distribution of PEP administration by species of biting animal. However, our tools could potentially be used to assess more than 300 000 emergency department bite events each year,^5^ leading to better informed decisions on the need to initiate the vaccination series.

Additionally, PEP recommendations could be improved if clinicians used health department rabies consultation services more frequently. In 2020, Steinberg et al^15^ found patients in Cook County, Illinois who received PEP were 87% less likely to have received inappropriate treatment if their health care practitioner consulted a health department (adjusted odds ratio, 0.13; 95% CI, 0.08-0.22). They also found differences in inappropriate PEP administration by animal species, which could be due to Advisory Committee on Immunization Practices guidelines being more complicated for domestic vs wild animals.^15^

### Limitations

An assumption of our model is that animal health status and exposure circumstances are independent. To avoid this assumption, we would need data on how those parameters vary together. These data exist for other countries (for example, through the Rabies Exposure Assessment and Contact Tracing App [REACT])^40^; however, the distribution of animals in those countries that use REACT App, such as Haiti and Vietnam, are heavily skewed toward dogs and may not be generalizable to the US. Additionally, our estimates do not apply to situations where the person was potentially exposed to a bat but had no known contact with the bat. More careful consideration is due for these circumstances as bites or scratches from a bat can go unnoticed or be trivialized due to the small size of their teeth.^7^

Although we know that unprovoked bite events from apparently ill animals increase the suspicion for rabies, the quantifiable increase in risk has not been described in North American wildlife. These key risk assessment variables are rarely reported to the NRSS; therefore, the data used to obtain the estimates for these 2 risk assessment criteria could be biased due to lack of geographic and species representativeness. Also, evaluation of health status and circumstances of the exposure often requires the expertise of a public health professional who is familiar with veterinary medicine and animal behavior. Misclassification of these variables by laypeople is common and could result in miscalculation of risk. Indeed, when there is uncertainty surrounding these variables, users of the tools should select the riskier response.

Furthermore, there is likely bias in some estimates due to the probability that certain species are more (or less) frequently captured and submitted for RABV testing. Animals that are considered low risk and cause fairly atraumatic bite wounds are likely only submitted for testing if there is a strong suspicion for RABV infection. For example, most health authorities would rarely recommend rabies testing or PEP after a bite from a small rodent.^8,9,11,41^ Our model estimates are consistent with this guidance for normal-acting rodents; however, if the small rodent is considered strange-acting and the bite was unprovoked, the model would suggest that PEP is recommended. This discrepancy highlights the importance of rabies experts to interpret the exposure scenario before initiating PEP and considering a higher threshold for “health status” and “provoked” for low-risk species. This potential bias also likely explains the moderate correlation between survey responses and Pr(rabid|exposure) (along with individual variation in experience and risk tolerance).

## Conclusions

Our model is based on more than 900 000 rabies case investigations. It is also the first national tool, to our knowledge, to account for geographic differences in the rabies risk associated with animal exposures. If implemented correctly, this tool has the potential to prevent unnecessary expenditures and adverse events for individual patients and improve the overall efficiency of PEP delivery in the US.

## Notes

Supplement 1. **eAppendix 1.** US Jurisdictional Abbreviations
eMethods.
**eFigure 1. **Flow Chart Showing Exclusion Criteria and Number of Rabies Virus (RABV) Samples Included in the Analysis, 2011 to 2020, US **eTable 1. **Animal Categories for Rabies Risk Model **eFigure 2. **Temporal Trends in Positivity Rates of Rabies Virus Across Animal Categories, 2011 to 2020 **eFigure 3. **Positivity Rates of Rabies Virus for Bats, Cats, and Dogs by US Jurisdiction, 2011 to 2020 **eFigure 4. **Positivity Rates of Rabies Virus for Raccoons, Skunks, and Foxes by US Jurisdiction, 2011 to 2020 **eFigure 5. **Positivity Rates of Rabies Virus (RABV) for Cattle, Equine, Hoofed Animals, Rodents of Unusual Size (ROUS), and Sheep and Goats, 2011 to 2020 **eFigure 6. **Positivity Rates of Rabies Virus by Bat Species, 2011 to 2020 **eFigure 7. **Pr(rabid|exposure) Associated With Survey Scenarios **eAppendix 2. **National Association of State Public Health Veterinarians Rabies Postexposure Prophylaxis Recommendation Questionnaire **eAppendix 3.** Rabies Risk Assessment Case Studies **eFigure 8. **Risk Thresholds (Vertical Colored/Dashed Lines) Associated With Each Value of the Decision Boundary, P, Considered in 10-Fold Cross-Validation **eTable 2. **Evaluation of the Decision Boundary for Logistic Regression Using 10-Fold Cross-Validation **eFigure 9. **Violin Plots of the Estimates From the Probability Model, US
eResults
**eFigure 10. **Probability Distributions for Developing Rabies From Single and Multiple Bite Exposures **eTable 3. **Estimates of the Probability of Developing Rabies According to Anatomical Location and Severity of Exposure From the Literature **eTable 4. **Final Parameter Estimates for Anatomical Location and Severity of Exposure **eFigure 11. **Spatial Distribution of Completed Surveys **eFigure 12. **Risk Profile of Survey Respondents According to Years of Experience **eTable 5. **Survey Scenarios Above the Estimated Threshold for Which Postexposure Prophylaxis Was Not Recommended (“Strongly Disagree,” N = 27) **eFigure 13. **Logistic Regression Diagnostics
eReferences

Supplement 2.
Data Sharing Statement
