# Supplementary material for: Risk of Rabies and Implications for Postexposure Prophylaxis Administration in the US
Source: JAMA Netw Open. 2023 Jun 9;6(6):e2317121. doi: 10.1001/jamanetworkopen.2023.17121 (PMC10257100; doi:10.1001/jamanetworkopen.2023.17121)
Supplement: Supplement 1. — eAppendix 1. US Jurisdictional Abbreviations eMethods. eFigure 1. Flow Chart Showing Exclusion Criteria and Number of Rabies Virus (RABV) Samples Included in the Analysis, 2011 to 2020, US eTable 1. Animal Categories for Rabies Risk Model eFigure 2. Temporal Trends in Positivity Rates of Rabies Virus Across Animal Categories, 2011 to 2020 eFigure 3. Positivity Rates of Rabies Virus for Bats, Cats, and Dogs by US Jurisdiction, 2011 to 2020 eFigure 4. Positivity Rates of Rabies Virus for Raccoons, Skunks, and Foxes by US Jurisdiction, 2011 to 2020 eFigure 5. Positivity Rates of Rabies Virus (RABV) for Cattle, Equine, Hoofed Animals, Rodents of Unusual Size (ROUS), and Sheep and Goats, 2011 to 2020 eFigure 6. Positivity Rates of Rabies Virus by Bat Species, 2011 to 2020 eFigure 7. Pr(rabid|exposure) Associated With Survey Scenarios eAppendix 2. National Association of State Public Health Veterinarians Rabies Postexposure Prophylaxis Recommendation Questionnaire eAppendix 3. Rabies Risk Assessment Case Studies eFigure 8. Risk Thresholds (Vertical Colored/Dashed Lines) Associated With Each Value of the Decision Boundary, P, Considered in 10-Fold Cross-Validation eTable 2. Evaluation of the Decision Boundary for Logistic Regression Using 10-Fold Cross-Validation eFigure 9. Violin Plots of the Estimates From the Probability Model, US eResults eFigure 10. Probability Distributions for Developing Rabies From Single and Multiple Bite Exposures eTable 3. Estimates of the Probability of Developing Rabies According to Anatomical Location and Severity of Exposure From the Literature eTable 4. Final Parameter Estimates for Anatomical Location and Severity of Exposure eFigure 11. Spatial Distribution of Completed Surveys eFigure 12. Risk Profile of Survey Respondents According to Years of Experience eTable 5. Survey Scenarios Above the Estimated Threshold for Which Postexposure Prophylaxis Was Not Recommended (“Strongly Disagree,” N = 27) eFigure 13. Logistic Regression Diagnostics [file jamanetwopen-e2317121-s001.pdf]

Supplemental Online Content

Charniga K, Nakazawa Y, Brown J, Jeon S, Wallace RM. Risk of rabies and implications for postexposure prophylaxis administration in the US. *JAMA Netw Open*. 2023;6(4):e2317121. doi:10.1001/jamanetworkopen.2023.17121

eAppendix 1. US Jurisdictional Abbreviations

eMethods

eFigure 1. Flow Chart Showing Exclusion Criteria and Number of Rabies Virus (RABV) Samples Included in the Analysis, 2011 to 2020, US

eTable 1. Animal Categories for Rabies Risk Model

eFigure 2. Temporal Trends in Positivity Rates of Rabies Virus Across Animal Categories, 2011 to 2020

eFigure 3. Positivity Rates of Rabies Virus for Bats, Cats, and Dogs by US Jurisdiction, 2011 to 2020

eFigure 4. Positivity Rates of Rabies Virus for Raccoons, Skunks, and Foxes by US Jurisdiction, 2011 to 2020

eFigure 5. Positivity Rates of Rabies Virus (RABV) for Cattle, Equine, Hoofed Animals, Rodents of Unusual Size (ROUS), and Sheep and Goats, 2011 to 2020

eFigure 6. Positivity Rates of Rabies Virus by Bat Species, 2011 to 2020

eFigure 7. Pr(rabid|exposure) Associated With Survey Scenarios

eAppendix 2. National Association of State Public Health Veterinarians Rabies Postexposure Prophylaxis Recommendation Questionnaire

eAppendix 3. Rabies Risk Assessment Case Studies

eFigure 8. Risk Thresholds (Vertical Colored/Dashed Lines) Associated With Each Value of the Decision Boundary, P, Considered in 10-Fold Cross-Validation

eTable 2. Evaluation of the Decision Boundary for Logistic Regression Using 10-Fold Cross-Validation

eFigure 9. Violin Plots of the Estimates From the Probability Model, US

eResults

eFigure 10. Probability Distributions for Developing Rabies From Single and Multiple Bite Exposures

© 2023 Charniga K et al. *JAMA Network Open.*

eTable 3. Estimates of the Probability of Developing Rabies According to Anatomical Location and Severity of Exposure From the Literature

eTable 4. Final Parameter Estimates for Anatomical Location and Severity of Exposure

eFigure 11. Spatial Distribution of Completed Surveys

eFigure 12. Risk Profile of Survey Respondents According to Years of Experience

eTable 5. Survey Scenarios Above the Estimated Threshold for Which Postexposure Prophylaxis Was Not Recommended (“Strongly Disagree,” N = 27)

eFigure 13. Logistic Regression Diagnostics

eReferences

This supplemental material has been provided by the authors to give readers additional information about their work.

© 2023 Charniga K et al. *JAMA Network Open.*

eAppendix 1. US Jurisdictional Abbreviations

Jurisdiction name	Abbreviation
Alabama	AL
Alaska	AK
Arizona	AZ
Arkansas	AR
California	CA
Colorado	CO
Connecticut	CT
District of Columbia	DC
Delaware	DE
Florida	FL
Georgia	GA
Hawaii	HI
Idaho	ID
Illinois	IL
Indiana	IN
Iowa	IA
Kansas	KS
Kentucky	KY
Louisiana	LA
Maine	ME
Maryland	MD
Massachusetts	MA
Michigan	MI
Minnesota	MN
Mississippi	MS
Missouri	MO
Montana	MT
Nebraska	NE
Nevada	NV
New Hampshire	NH
New Jersey	NJ
New Mexico	NM
New York	NY
North Carolina	NC
North Dakota	ND
Ohio	OH
Oklahoma	OK
Oregon	OR
Pennsylvania	PA
Puerto Rico	PR
Rhode Island	RI
South Carolina	SC
South Dakota	SD
Tennessee	TN
Texas	TX
Utah	UT

© 2023 Charniga K et al. *JAMA Network Open.*

Vermont	VT
Virginia	VA
Washington	WA
West Virginia	WV
Wisconsin	WI
Wyoming	WY

© 2023 Charniga K et al. *JAMA Network Open.*

eMethods

Surveillance data. Since 1944, rabies has been a nationally notifiable condition in the United States for humans and animals.[1,2] Laboratory-confirmed cases are reported to state public health officials and the National Rabies Surveillance System (NRSS), which is maintained by the Poxvirus and Rabies Branch within the U.S. Centers for Disease Control and Prevention (CDC). Data are submitted to the NRSS from a network of 125 state public health, agriculture, and university laboratories which carry out the direct fluorescent antibody test for rabies virus (RABV).[3,4] The direct fluorescent antibody test is considered the gold standard for rabies diagnostic testing with sensitivity and specificity near 100%.[5] Although most samples are submitted via passive surveillance (i.e., if a person or animal is exposed to a potentially rabid animal), active surveillance activities are also performed by the U.S. Department of Agriculture (USDA) Wildlife Services. The Council of State and Territorial Epidemiologists suggests including nine variables in all reports of rabies in animals, such as species, location of animal capture, and whether humans or other animals were exposed.[6]

There were 984,159 samples submitted for RABV testing that were reported to CDC between 2011 – 2020 (1 brain sample per animal, **eFigure 1**). We excluded samples that were collected through active surveillance (e.g., typically not associated with a human or other animal exposure). We also excluded samples with indeterminate RABV test results and those that were not ultimately tested. When possible, we used the jurisdiction corresponding to the origin of the animal, not where the animal was reported. Samples with "out of state" and "unknown" location were excluded as well as those in which the animal originated in Canada, Hawaii (which is rabies-free), and the Virgin Islands. Samples from New York City were combined with those from New York. Samples corresponding to animal types that were ambiguous or did not fit into other categories were excluded (e.g., humans, hybrids, and marsupials) along with those that do not transmit the virus (non-mammals, e.g., birds). There were 935,881 samples remaining for analysis from 51 jurisdictions (49 states plus Washington D.C. and Puerto Rico) (**eTable 1**). The number of samples each year ranged from 77,904 in 2020 to 101,693 in 2014.

Classifying animals. We grouped armadillo and opossum together because although they differ taxonomically,[7] they share similar diets consisting mainly of insects and plants.[8] Also, some jurisdictions, including Florida and Texas,[9,10] classify both animals as low risk for rabies. We created a separate category for bobcats rather than group them with large carnivores because there were many samples (N = 1,088, **eTable1**) and some jurisdictions, such as Florida,[9] have specific recommendations about this animal being a high-risk species.

Bat species. Forty-two percent (N = 106,501) of 253,028 bat samples in the data had information about the species. We removed vampire bats and mega bats which are not currently found in the wild in the United States (N = 55, representing 4 species). We also removed 17 species with high uncertainty in positivity rates due to small sample sizes (95% exact binomial confidence intervals of positivity rates greater than or equal to 0.25), resulting in 106,346 samples across 28 species.

Positivity rates. Positivity rates will be higher than the RABV infection prevalence in the general animal population as the samples are submitted via passive surveillance. For bats, cats, and dogs, we calculated jurisdiction-specific positivity rates because sufficient data were available across all jurisdictions (**eFigure 3**). We also calculated jurisdiction-specific rates for raccoons, skunks, foxes, arctic foxes (Alaska only), and the small Indian mongoose (Puerto Rico only) because distinct RABV variants are associated with these reservoir species (**eFigure 4**). For jurisdictions with high uncertainty of positivity rates for certain animals (the width of the 95% exact binomial confidence intervals was greater than or equal to 0.25), we used regional, rather than jurisdiction-specific, estimates. The regions were defined according to the primary rabies reservoir in each jurisdiction (skunk, bat, or raccoon).[4]

We calculated two regional positivity rates each for cattle, equine, sheep and goats, rodents of unusual size, and hoofed animals, pooling samples across jurisdictions according to whether terrestrial RABV variants are present (**eFigure 5**). Rabies risk is considered higher for these species in states where terrestrial RABV variants are found. We classified Washington, Oregon, Nevada, Idaho, Illinois, Indiana, Mississippi, and Utah as being free from terrestrial RABV.[4]

For the remaining animal categories, we pooled samples across all jurisdictions to calculate national estimates of the positivity rates (**eTable 1**). We also calculated positivity rates for bat species (**eFigure 6**).

© 2023 Charniga K et al. *JAMA Network Open.*

Parameter estimates for health status. New Hampshire and Arkansas were the only two jurisdictions that provided data regarding an animal's health status. New Hampshire had 489 and 506 samples with a conclusive (positive or negative) test result for 2018 and 2020, respectively. For those years, presence of rabies symptoms was reported as a binary variable (yes/no). A list of symptoms (what constituted a "yes") was not provided with the data. For 2018, only 94 samples had non-missing symptom information, and for 2020, 84 samples had non-missing symptom information, for a total of 178 samples. Eight out of 178 total samples had reported symptoms.

In 2020, Arkansas had 676 samples with a positive or negative test result. Of those, symptom information was reported (non-missing) for 238 animals as free text. We removed 44 samples in which the circumstances of the exposure were reported rather than symptoms (e.g., hit by car or found dead). For the remaining 194 samples, we separated animals that were apparently healthy from those that were ill or acting strangely by searching the free text using the following key words: "none," "no symptoms," and "healthy." Samples that were classified as ill or acting strangely had words including "out during day" (for nocturnal species), "slobbering," "staggering", "paralysis", and "unusual viciousness," among others.

We included USDA data for animal health status because they contain a larger proportion of healthy animals compared to data from the jurisdictions due to active surveillance activities, which include trapping and euthanasia regardless of human or other animal exposure (i.e., to pets). From 2015 – 2020, 41,716 samples with a positive or negative test result were reported by USDA. Of those, 2,750 had non-missing information for the variable "why euthanized"; 412 animals were euthanized for "abnormal behavior," while 2,338 exhibited "normal behavior."

We combined the data from New Hampshire, Arkansas, and USDA and constructed a 2x2 table of health status by RABV test result.

Parameter estimates for exposure circumstances. Similarly, Arizona was the only jurisdiction that reported data on the circumstances of the exposure for its RABV samples. Arizona reported information about the circumstance of exposure for RABV samples submitted from 2018 – 2020. A positive or negative test result was reported for 618, 562, and 440 samples in 2018, 2019, and 2020, respectively. We restricted the data to only include instances where "yes" was selected for the variable "was there possible human exposure" and the number of humans with possible exposure was at least 1. We then excluded samples for which the variable "was the contact bite provoked" was missing, resulting in 188 samples for 2018, 186 for 2019, and 122 for 2020. Of the 496 total samples, 358 exposures were provoked, while 138 were unprovoked. We constructed a 2x2 table of exposure circumstance by RABV test result.

Literature review. We began our review by searching Google Scholar for the terms "rabies" AND "death" AND "probability" on October 19, 2021. We checked the references of relevant publications to identify as many original data sources as possible. We limited our search mainly to English-language publications except for one key reference, which was written in French. Articles were excluded if they did not report the probability of death from rabies by anatomical location and severity of exposure. We updated our search on April 11, 2023 using the following additional search terms: "virus" and "animal." We also expanded the search to PubMed but did not find any additional publications.

We expected to find few, if any, publications from the United States as there have only been 1 – 2 human rabies deaths per year since 1960.[11]

Survey. Survey data were collected online through Google Forms. A copy of the survey is included in **Part 3**. Respondents were asked whether they agreed with the following statement about a possible RABV exposure in their jurisdiction: post-exposure prophylaxis (PEP) is recommended. They could select from four options: strongly agree, agree, disagree, and strongly disagree. Only the following information was provided about each scenario:

- Animal species/common name
- Whether the exposure was provoked
- Whether the animal is healthy
- Whether the animal is up to date on its rabies vaccines (domestic animals only)

Survey scenarios were selected to represent a range of risk probabilities; however, scenarios with lower probabilities were overrepresented to estimate a risk threshold (**eFigure 7**). Seven questions asked about a scenario with a probability corresponding to a pooled, national estimate. These included: small rodents, lagomorphs, and Eulipotyphla; ferret; armadillo and opossum; non-native wild; mesocarnivores; bobcat; and swine. We did not present the results of the final question about the urgency with which PEP should be administered given that an exposure had occurred and PEP had been recommended, as some respondents found it confusing.

We first piloted the survey among CDC's Rabies Epidemiology Team (N = 9). Seven team members conduct >1,500 rabies consultations each year through a public-facing hotline and email box. Each member took the survey twice, first representing Georgia and second representing another jurisdiction. The average completion time of the survey was 12 minutes in the pilot study. We performed a preliminary analysis of the pilot survey data to inform the larger survey (results not shown).

For the real survey, the target sample size was at least two respondents from each state, except for Hawaii, plus Washington D.C. and Puerto Rico (N = 102). We wanted to get good geographic coverage and recognize that only 1 – 2 individuals work on rabies at the state health department level per jurisdiction. Ideally, these individuals would have at least five years of experience making professional recommendations regarding rabies PEP. The NASPHV Executive Board emailed the survey link to state public health partners, and data collection was open from May 24, 2022 – August 5, 2022.

Cleaning survey data. We had 1,200 observations (24 scenarios * 50 respondents) after matching the scenarios with the quantitative estimates; however, 6 observations were removed from Puerto Rico involving skunks, raccoons, and foxes because no samples were available for those animals in the surveillance data from 2011 – 2020. Alternatively, we could have assumed Puerto Rico had the same risk for these scenarios as Florida due to similar climate and geographic proximity. See eResults for details about the survey respondents.

Accounting for uncertainty for animals with no reported positive cases. To capture uncertainty in the data for animal categories with positivity rates of 0, such as pocket pets, we used the fact that a beta distribution is a conjugate prior distribution for the binomial distribution.[12] A prior distribution and likelihood function are considered conjugate if by multiplying them together and rescaling, a posterior distribution in the same family as the prior distribution is obtained. We combined the binomial likelihood with a beta(1,1), or uniform prior distribution, to obtain a beta posterior distribution and then sampled from the posterior distribution.[12]

Determining the decision boundary for p in logistic regression. Choosing p, the threshold for the decision in logistic regression, depends on the prediction context and is therefore subjective. In this analysis, p represents whether PEP would be recommended for different Pr(rabid|exposure) based on subject matter experts' opinion from the survey. We performed 10-fold cross-validation and selected the decision boundary with the highest F-score. F-score is the harmonic mean of precision and recall (sensitivity) and is considered the best performance metric to use with unbalanced data.[13]

We randomly split the survey data into 10 equally sized folds. For each fold, we trained the logistic regression model on the remaining 9 folds (as in the main text, with the natural log of Pr(rabid|exposure) as the predictor and PEP yes vs no as the outcome) and saved the model coefficients. Then we used the coefficients to make predictions on the fold that was not used to train the model (the testing set). We saved the number of false positives (FP), false negatives (FN), and true positives (TP) corresponding to decision boundaries ranging from 0.35 to 0.75. We summed the FPs, FNs, and TPs across the 10 folds and calculated the F-score as (2 * TP) / (2 * TP + FP + FN) for each decision boundary. This approach to calculating F-score is preferred over calculating the mean of F-score across the folds because it avoids the problem of undefined precision or recall.[13]

Sensitivity analyses on survey data. We repeated the analysis of the survey data by only including respondents with at least 9 years of professional experience recommending PEP (N = 22 experts and 528 observations).

As survey responses consisting of "agree" and "disagree" may be more subjective than "strongly agree" and "strongly disagree", we also repeated the logistic regression analysis of the survey data using only "strongly agree" (N = 499) and "strongly disagree" (N = 61) responses.

We investigated survey scenarios in which respondents selected “disagree” or “strongly disagree” for PEP, but the Pr(rabid|exposure) was above our estimated threshold. These scenarios represent false positives in which our analysis suggests PEP is needed, but experts would not recommend it in practice.

Shiny apps. The first Shiny app we developed applies to all animal exposures in the United States, while the second applies only to bat species. We provided the risk threshold in the apps to interpret the estimates for Pr(rabid|exposure). For Pr(death|exposure), users can compare the risks for possible RABV exposures to the probability of dying within one year as estimated by the U.S. Social Security Administration by age and sex.[14] We used the 2019 period life table, which was used in the 2022 Trustees Report. We also compared Pr(death|exposure) to the lifetime probabilities of dying from other causes. The National Safety Council publishes the lifetime odds of death for selected causes in the United States.[15] We used the most current estimates, which were calculated using the population and life expectancy for the year 2020, and converted the odds to probabilities.

The two Shiny apps work by filtering down a dataset based on the user’s selections. Options include U.S. jurisdiction, animal category, provoked vs unprovoked exposure, animal being apparently healthy vs ill or acting strangely, and animal’s vaccination status. The bat species app is agnostic to geographic location and was requested by CDC partners at the National Park Service. Even if a bat is unavailable, sometimes park rangers can guess the species based on the specific geographic location of the exposure in the national parks. The bat species tool was also created because most human rabies deaths in the United States are attributed to bat exposures, and certain species are known to be high-risk for rabies.[16]

Number needed to treat (NNT). Assuming PEP reduces the risk of rabies death by 100%,[17] NNT = 1/Pr(death|exposure). We calculated NNT using the median estimates of Pr(death|exposure). To obtain the PEP cost per death averted, we multiplied NNT by $3,800, the estimated average cost of PEP.[18]

© 2023 Charniga K et al. *JAMA Network Open.*

eFigure 1. Flow Chart Showing Exclusion Criteria and Number of Rabies Virus (RABV) Samples Included in the Analysis, 2011 to 2020, US

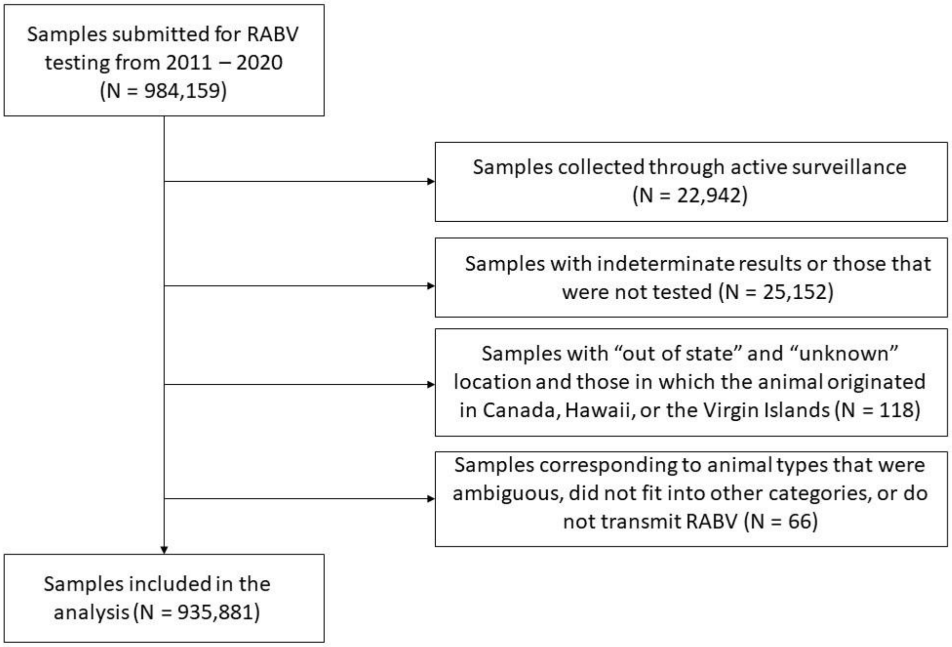

© 2023 Charniga K et al. *JAMA Network Open.*

eTable 1. Animal Categories for Rabies Risk Model

Animals were classified into 23 mutually-exclusive risk groups for rabies based on existing guidance, taxonomy, diet, and geographical location (native vs non-native wild). 935,881 total samples submitted for rabies virus testing were available for analysis from 2011 – 2020.

Category	Animal types	Number of samples	Positivity rate (95% CI)[a]
Bat		253,028	0.062 (0.061 – 0.062)
Cat		222,314	0.012 (0.011 – 0.012)
Dog		220,960	0.0030 (0.0027 – 0.0032)
Raccoon		109,943	0.15 (0.14 – 0.15)
Skunk		41,859	0.29 (0.29 – 0.30)
Fox		16,548	0.20 (0.20 – 0.21)
Small rodents, lagomorphs, and Eulipotyphla[b]	Chipmunk, gopher, lagomorph, mole, mouse, muskrat, prairie dog, rabbit, rat, rodent, shrew, squirrel, flying squirrel, vole	12,065	0.0008 (0.0004 – 0.0015)
Cattle		11,491	0.056 (0.052 – 0.061)
Rodents of unusual size	Beaver, groundhog, marmot, nutria, porcupine	9,480	0.041 (0.037 – 0.045)
Mesocarnivores[c]	Coyote, badger, wolverine, otter, coati, lynx, ermine, mink, fisher, marten, weasel, ringtail	8,996	0.018 (0.016 – 0.021)
Equine	Horse, equine, donkey, mule, miniature donkey	7,658	0.032 (0.028 – 0.036)
Armadillo and opossum		6,640	0.003 (0.002 – 0.004)
Sheep and goats		5,600	0.019 (0.015 – 0.022)
Hoofed animals[d]	Deer, elk, moose, antelope, pronghorn, bison, Asian water buffalo, reindeer, muskox, bighorn sheep, javelina	2,802	0.022 (0.017 – 0.029)
Unknown or not provided		1,661	0.004 (0.001 – 0.008)
Bobcat		1,088	0.13 (0.11 – 0.15)
Non-native wild	African wild dog, binturong, kudu, serval, tapir, cheetah, rhinoceros, tiger, kangaroo, zebra, caracal, wallaby, leopard, giraffe, Siberian lynx, red panda, llama, lion, chimpanzee, alpaca, jaguar, monkey, macaque, gazelle, camel, camelid, kinkajou, yak, marmoset, meerkat, capybara, hippopotamus, ocelot, oryx, eland, lemur, hyrax, wallaroo, genet	878	0.011 (0.005 – 0.021)
Large carnivores	Bear, cougar, sea lion, seal, wolf, mountain lion, panther, puma	826	0.011 (0.005 – 0.021)
Ferret		593	0.005 (0.001 – 0.015)
Swine		583	0.010 (0.004 – 0.022)
Arctic fox[e]		379	0.11 (0.08 – 0.15)
Mongoose[f]		279	0.83 (0.78 – 0.87)
Pocket pets	Chinchilla, gerbil, guinea pig, hamster, hedgehog, sugar glider	210	0 (0 – 0.017)

[a]Exact binomial confidence intervals

© 2023 Charniga K et al. *JAMA Network Open.*

[b]Moles and shrews are not rodents; they are classified in the order Eulipotyphla
[c]Eat about 50% meat
[d]Herbivores
[e]Alaska only
[f]Puerto Rico only

© 2023 Charniga K et al. *JAMA Network Open.*

eFigure 2. Temporal Trends in Positivity Rates of Rabies Virus Across Animal Categories, 2011 to 2020

Positivity rates were calculated from passive surveillance data as the number of samples that tested positive divided by the total number of samples with a positive or negative test result. The total number of samples is shown in each plot. See eTable 1 for the list of species that make up these categories.

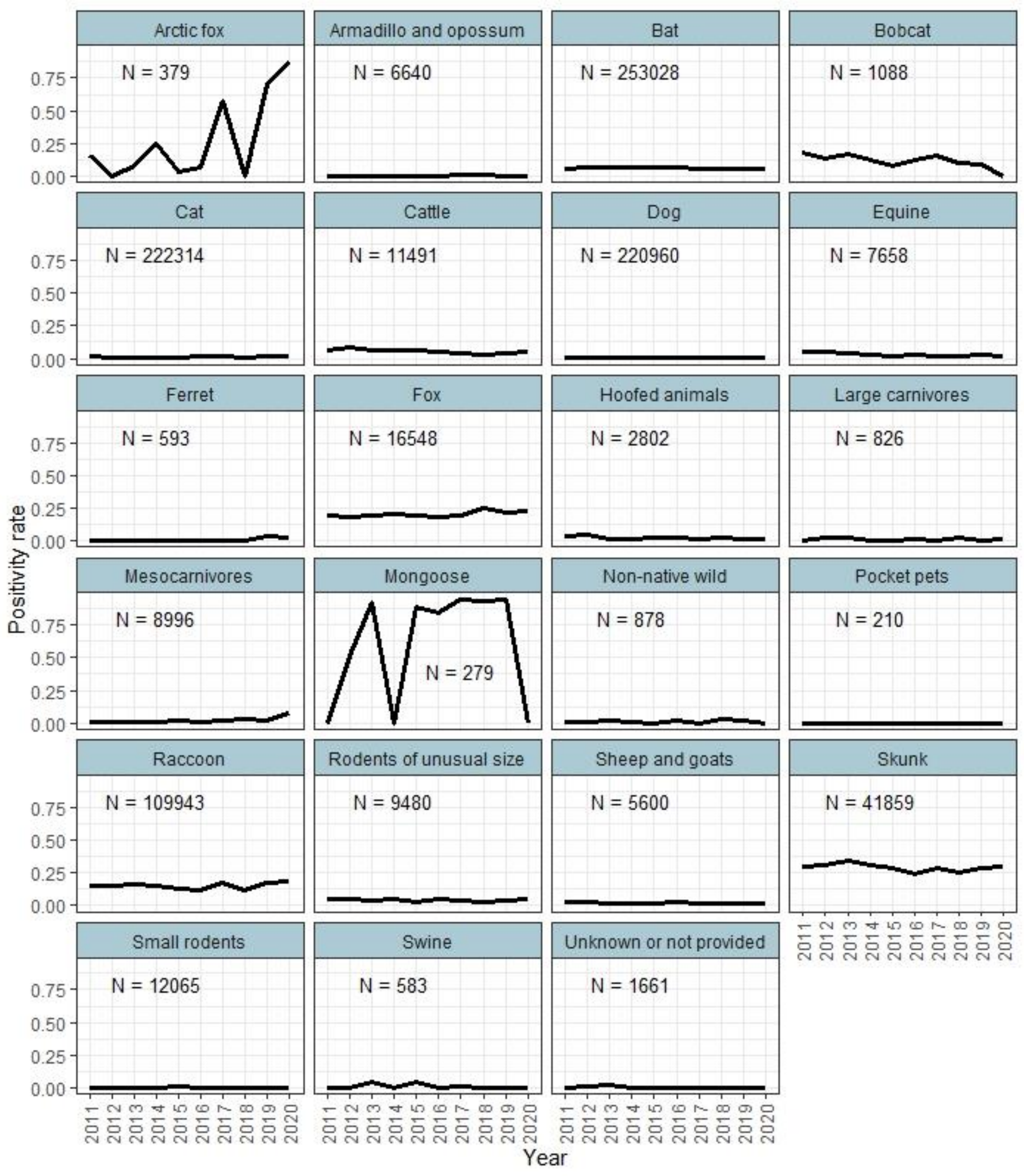

© 2023 Charniga K et al. *JAMA Network Open.*

eFigure 3. Positivity Rates of Rabies Virus for Bats, Cats, and Dogs by US Jurisdiction, 2011 to 2020

Color indicates main rabies virus reservoir in each jurisdiction. Positivity rates were calculated from passive surveillance data as the number of samples that tested positive divided by the total number of samples with a positive or negative test result. Median and 95% confidence interval (binomial exact test) are shown.

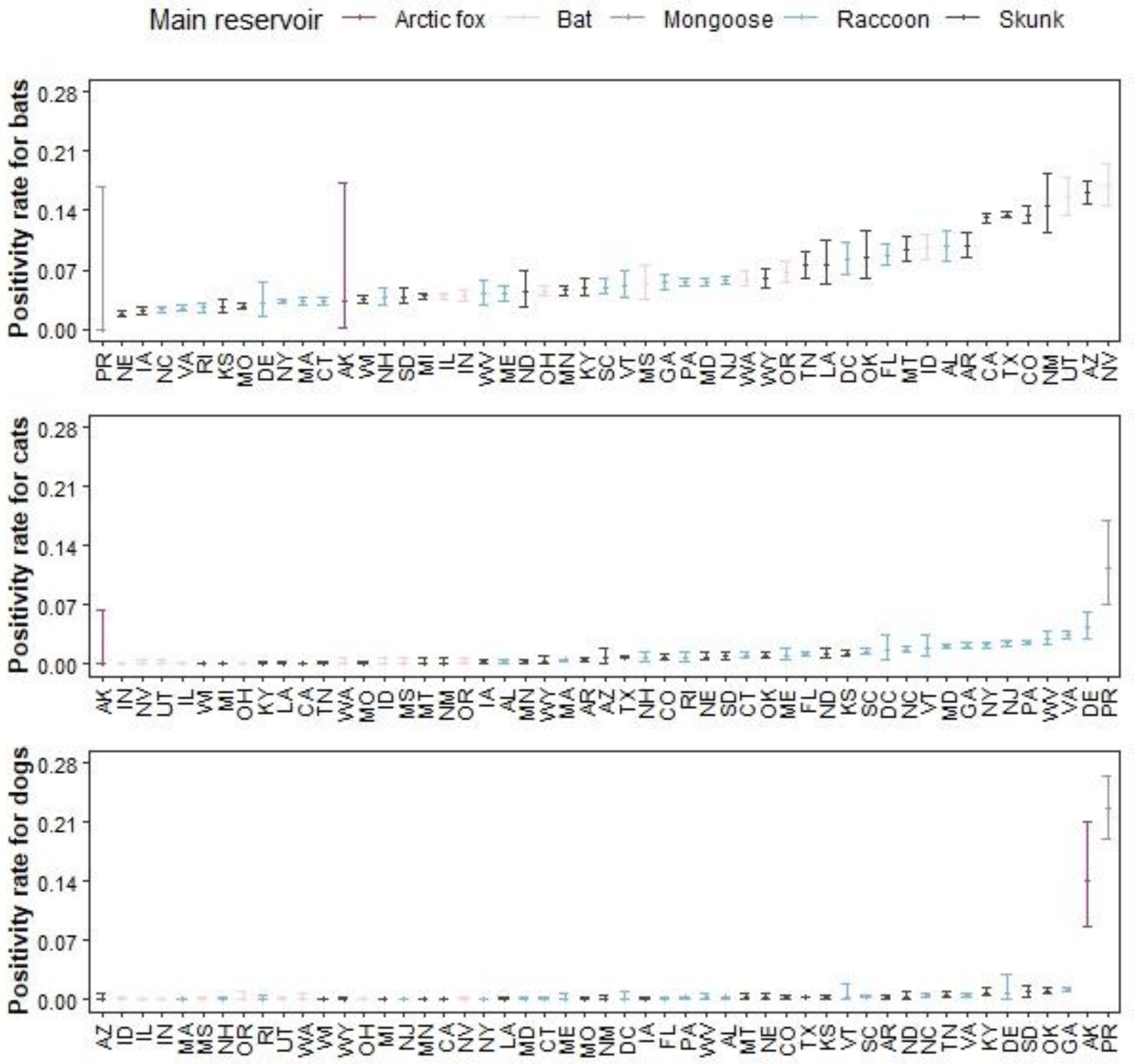

© 2023 Charniga K et al. *JAMA Network Open.*

eFigure 4. Positivity Rates of Rabies Virus for Raccoons, Skunks, and Foxes by US Jurisdiction, 2011 to 2020

Color indicates main rabies virus reservoir in each jurisdiction. A regional estimate of the positivity rate of skunks was used for five jurisdictions, and for foxes, a regional estimate was used for 10 jurisdictions; for these jurisdictions, the original estimate is shown by transparent points and error bars, while the regional estimate is shown by solid points and error bars. Positivity rates were calculated from passive surveillance data as the number of samples that tested positive divided by the total number of samples with a positive or negative test result. Median and 95% confidence interval (binomial exact test) are shown. From 2011 – 2020, no samples of raccoons, skunks, or foxes were available for Puerto Rico, while no samples of raccoons or foxes were available for Alaska.

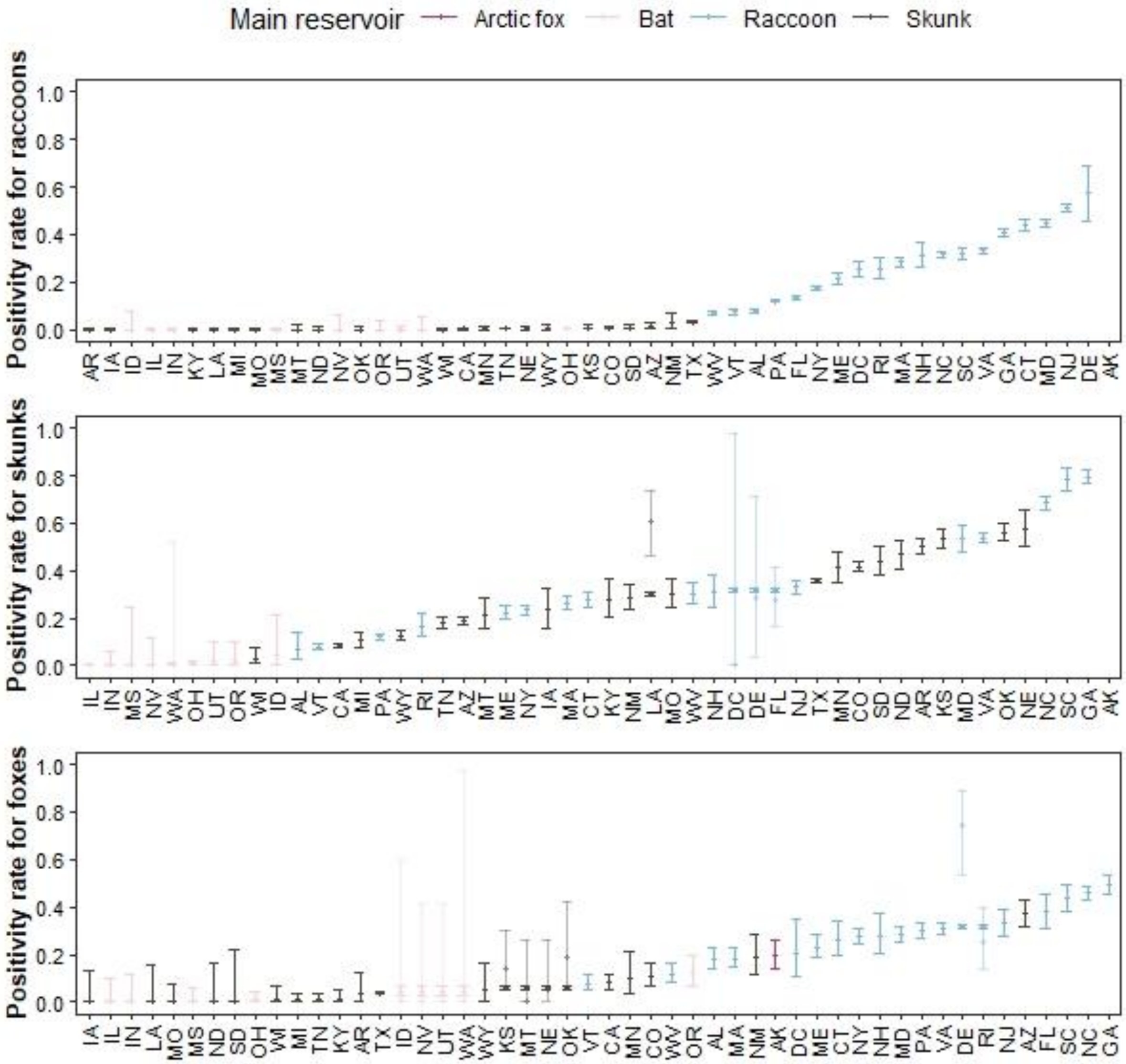

© 2023 Charniga K et al. *JAMA Network Open.*

eFigure 5. Positivity Rates of Rabies Virus (RABV) for Cattle, Equine, Hoofed Animals, Rodents of Unusual Size (ROUS), and Sheep and Goats, 2011 to 2020
Positivity rates were calculated by pooling samples across all jurisdictions ("National") as well as across jurisdictions according to the presence of terrestrial RABV variants. Positivity rates were calculated from passive surveillance data as the number of samples that tested positive divided by the total number of samples with a positive or negative test result. Median and 95% confidence interval (binomial exact test) are shown.

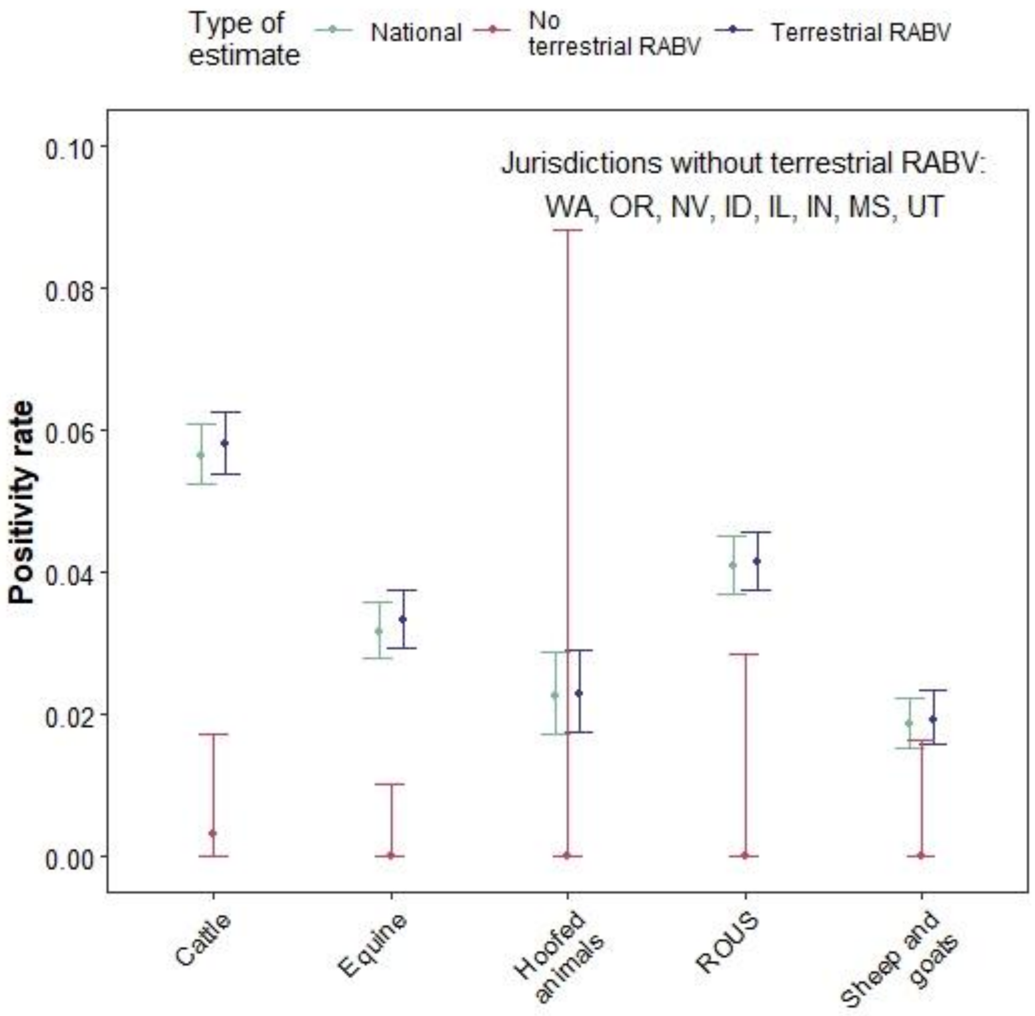

© 2023 Charniga K et al. *JAMA Network Open.*

eFigure 6. Positivity Rates of Rabies Virus by Bat Species, 2011 to 2020
Positivity rates were calculated as the number of samples that tested positive divided by the total number of samples with a positive or negative test result. Median and 95% confidence interval (binomial exact test) are shown. Positivity rates for all bats as well as those with unknown species are also shown.

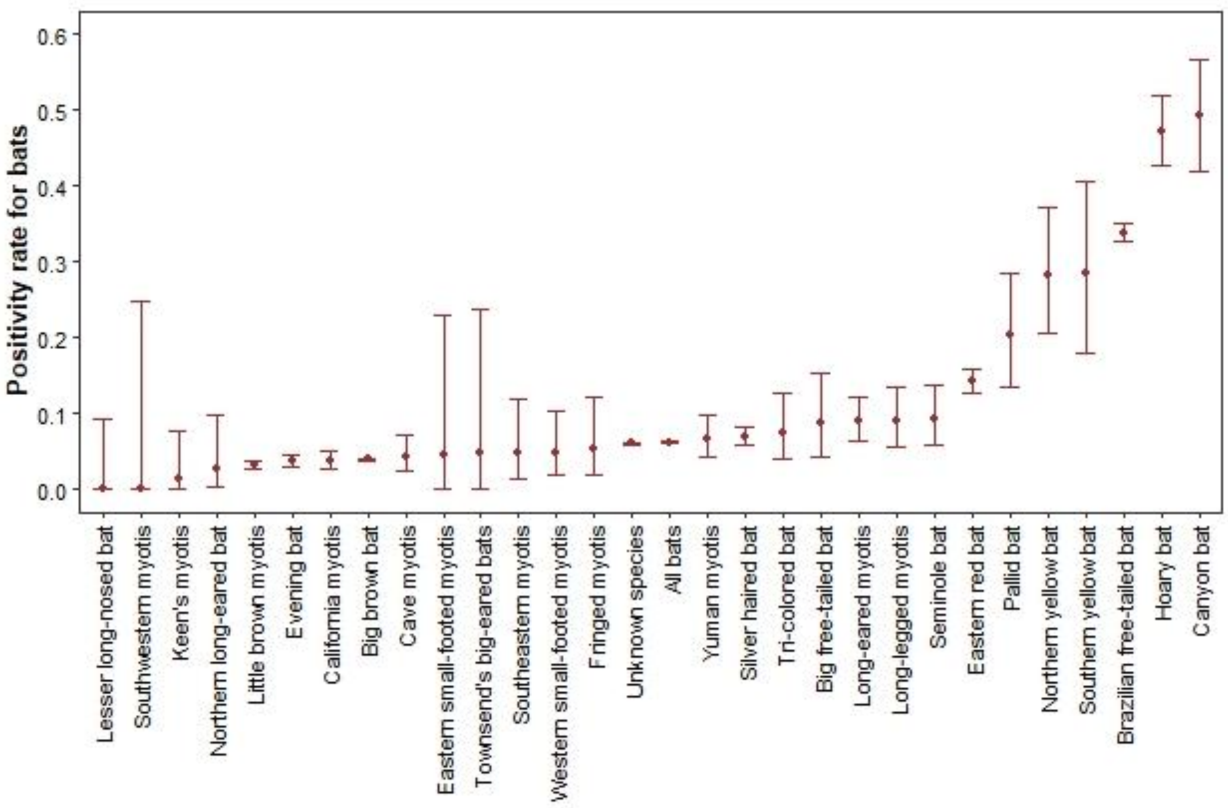

© 2023 Charniga K et al. *JAMA Network Open.*

eFigure 7. Pr(rabid|exposure) Associated With Survey Scenarios
Median and 95% credible intervals are shown for each scenario, which is represented on the x-axis. Scenarios were ordered by increasing Pr(rabid|exposure). As an example, the point on the far left of the plot represents the least risky scenario: an exposure to cat in Illinois that was apparently healthy, vaccinated, and provoked. Colors indicate whether the estimate was jurisdiction-specific or national.

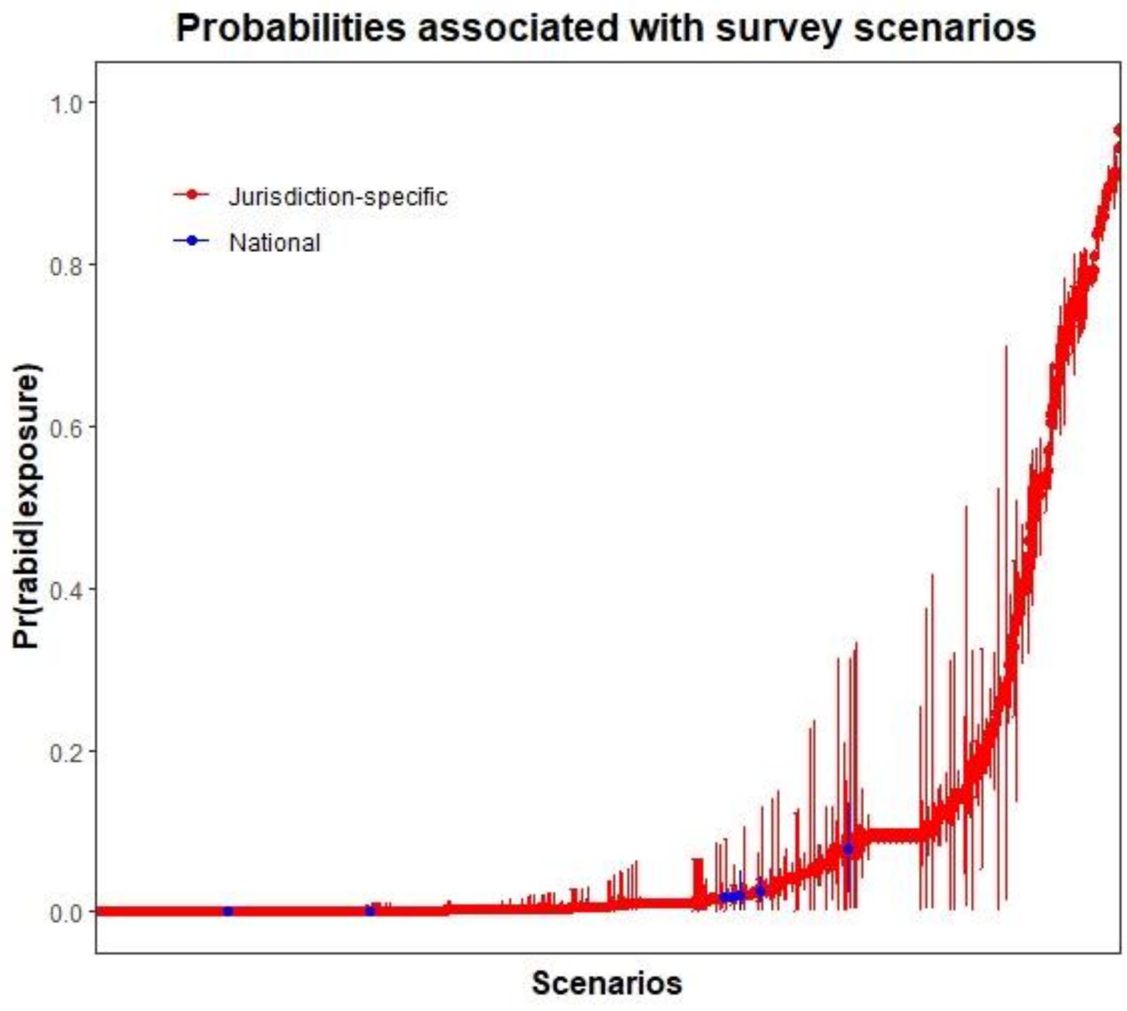

© 2023 Charniga K et al. *JAMA Network Open.*

eAppendix 2. National Association of State Public Health Veterinarians

Rabies Postexposure Prophylaxis Recommendation Questionnaire

For each scenario, please indicate whether you agree with the following statement: post-exposure prophylaxis (PEP) is recommended. Assume that the animal is not available for testing or observation, that an exposure has occurred, and that the exposure occurred in your state. Scenarios are presented in the following way: Animal species / whether the exposure was provoked / whether the animal is healthy / vaccination status of the animal (domestic animals only).

The final question asks about the urgency with which PEP should be administered given that an exposure has occurred and PEP has been recommended.

Name:

Email:

State:

At which level of public health do you work?
- National
- State
- County
- Local

Which option best describes your education or experience (select all that apply)?
- Clinical
- Epidemiology
- Veterinary medicine
- Other

Approximately how many years of experience making professional recommendations regarding rabies PEP do you have?

Scenario 1: Rat / unprovoked / apparently healthy
- Strongly agree
- Agree
- Disagree
- Strongly disagree

Scenario 2: Dog / provoked / ill or acting strangely / vaccinated
- Strongly agree
- Agree
- Disagree
- Strongly disagree

Scenario 3: Fox / unprovoked / apparently healthy
- Strongly agree
- Agree
- Disagree
- Strongly disagree

Scenario 4: Goat / provoked / apparently healthy / unvaccinated

© 2023 Charniga K et al. *JAMA Network Open.*

- Strongly agree
- Agree
- Disagree
- Strongly disagree

Scenario 5: Dog / unprovoked / apparently healthy / vaccinations not up to date
- Strongly agree
- Agree
- Disagree
- Strongly disagree

Scenario 6: Ferret / unprovoked / ill or acting strangely / unvaccinated
- Strongly agree
- Agree
- Disagree
- Strongly disagree

Scenario 7: Skunk / provoked / ill or acting strangely
- Strongly agree
- Agree
- Disagree
- Strongly disagree

Scenario 8: Bat / provoked / apparently healthy
- Strongly agree
- Agree
- Disagree
- Strongly disagree

Scenario 9: Cat / unprovoked / apparently healthy / vaccinated
- Strongly agree
- Agree
- Disagree
- Strongly disagree

Scenario 10: Horse / unprovoked / apparently healthy / unvaccinated
- Strongly agree
- Agree
- Disagree
- Strongly disagree

Scenario 11: Opossum / unprovoked / ill or acting strangely
- Strongly agree
- Agree
- Disagree
- Strongly disagree

Scenario 12: Cat / provoked / ill or acting strangely / vaccination status unknown
- Strongly agree
- Agree
- Disagree
- Strongly disagree

Scenario 13: Raccoon / unprovoked / ill or acting strangely

© 2023 Charniga K et al. *JAMA Network Open.*

- Strongly agree
- Agree
- Disagree
- Strongly disagree

Scenario 14: Bat / provoked / ill or acting strangely
- Strongly agree
- Agree
- Disagree
- Strongly disagree

Scenario 15: Deer / provoked / apparently healthy
- Strongly agree
- Agree
- Disagree
- Strongly disagree

Scenario 16: Dog / unprovoked / ill or acting strangely / vaccinated
- Strongly agree
- Agree
- Disagree
- Strongly disagree

Scenario 17: Raccoon / unprovoked / apparently healthy
- Strongly agree
- Agree
- Disagree
- Strongly disagree

Scenario 18: Skunk / unprovoked / ill or acting strangely
- Strongly agree
- Agree
- Disagree
- Strongly disagree

Scenario 19: Llama / provoked / ill or acting strangely
- Strongly agree
- Agree
- Disagree
- Strongly disagree

Scenario 20: Coyote / provoked / apparently healthy
- Strongly agree
- Agree
- Disagree
- Strongly disagree

Scenario 21: Fox / provoked / apparently healthy
- Strongly agree
- Agree
- Disagree
- Strongly disagree

Scenario 22: Bobcat / provoked / apparently healthy

- Strongly agree
- Agree
- Disagree
- Strongly disagree

Scenario 23: Beaver / provoked / ill or acting strangely

- Strongly agree
- Agree
- Disagree
- Strongly disagree

Scenario 24: Pig / unprovoked / ill or acting strangely / unvaccinated

- Strongly agree
- Agree
- Disagree
- Strongly disagree

© 2023 Charniga K et al. *JAMA Network Open.*

A patient has had a suspected rabies virus exposure and your health department has recommended PEP. If they go urgently to the Emergency Department (Day 1), the patient will have to pay $25,000 for treatment. If they coordinate PEP through other service providers, i.e., urgent care (Day 2), their family doctor (Day 3), local travel health clinic, etc. the costs decrease to $600 on Day 7. Assume that (i) the patient is uninsured and has a household income equal to the national median ($68,000) and (ii) the wound was thoroughly cleaned by the patient on Day 1. Based on the level of exposure, please indicate the urgency by which you'd recommend PEP considering the burden to the patient and the type of exposure. You can select the same day for more than one exposure type. [Please do not select more than one response per column.]

	Multiple bites / legs	Mucosal / head	Multiple bites / head	Lick to broken skin / arm	Single bite / torso	Single bite / arm	Single bite / leg	Scratch from animal's teeth / arm
Day 1: $25,000								
Day 2: $15,000								
Day 3: $6,500								
Day 4: $3,800								
Day 5: $1,200								
Day 6: $900								
Day 7: $600								

© 2023 Charniga K et al. *JAMA Network Open.*

eAppendix 3. Rabies Risk Assessment Case Studies

State and local public health officials often consult CDC's rabies epidemiology team on rabies exposures in their jurisdictions via phone or email. We collected examples of consults that occurred in 2022 and compared the model recommendation in each case with the actual decision that was made. Examples were also submitted by colleagues on the National Association of Public Health Veterinarian's (NASPHV) Executive Board.

Note that given the fatal nature of rabies, public health and clinical decisions are generally made based on an individual's probability of exposure, Pr(rabid|exposure), rather than their probability of dying from an exposure, Pr(death|exposure). Nevertheless, we provided both estimates for the scenarios in this supplement whenever possible.

Case study 1: New York sloth
Scenario. A child reached into a sloth's enclosure in New York and pulled its tail [provoked], prompting the sloth to scratch the child with its teeth. The sloth was apparently healthy but unvaccinated. The child was unable to be reached for post-exposure prophylaxis (PEP), and health officials needed to decide whether to kill and test the animal, which had been at the facility for four months (NASPHV recommends that wild-caught animals with public contact be quarantined for a minimum of six months).[27]
Estimates. Although no sloths had been tested for rabies virus (RABV) in the United States from 2011 – 2020, the animal would be classified as non-native wild, which is a pooled, national estimate. The Pr(rabid|exposure) was 0.0013 (95% CrI: 0.0005 – 0.0023), and Pr(death|exposure) was 0.00007 (95% CrI: 0.00003 – 0.00011) for the child, assuming the exposure was to the upper extremities.
Tool recommendation. Considering the uncertainty in the estimate, Pr(rabid|exposure) overlapped with the risk threshold of 0.0004 (95% CI: 0.0002 – 0.0006). Assuming the child was a 10-year-old male, the probability of dying within one year from any cause is estimated to be 0.000097,[14] which is higher than the median Pr(death|exposure) from the animal exposure. If the child could be located, PEP could be considered based on Pr(rabid|exposure); PEP would be recommended based on Pr(death|exposure).
Outcome. Health officials elected for a 30-day quarantine of the sloth.

Case study 2: Pennsylvania raccoon
Scenario. A woman in Pennsylvania found her grandchildren outside trying to "save" a raccoon that was showing neurological signs, including excessive salivation. The children's efforts included trying to give the raccoon water [provoked]. The raccoon was killed, and testing was inconclusive.
Estimates. The Pr(rabid|exposure) was 0.25 (95% CrI: 0.23 – 0.27). Not enough information was available to determine Pr(death|exposure).
Tool recommendation. If the grandchildren were exposed to the raccoon (the animal's saliva contact broken skin or mucous membranes), PEP would be recommended by the tool as the Pr(rabid|exposure) is 625 times the risk threshold.
Outcome. PEP was recommended.

Case study 3: Pennsylvania dog
Scenario. A dog presented to a veterinary clinic in Pennsylvania with acute pain (thought to be due to a kidney infection) and bit a veterinary technician on the hand [likely provoked if being handled]. The dog was not up to date on its rabies vaccinations, and the owner refused to provide information about how the dog was housed.
Estimates. The Pr(rabid|exposure) was 0.0054 (95% CrI: 0.0034 – 0.0077), and the Pr(death|exposure) was 0.0013 (95% CrI: 0.0007 – 0.0022).
Tool recommendation. The Pr(rabid|exposure) is above the risk threshold of 0.0004 (95% CI: 0.0002 – 0.0006). Assuming the veterinary technician was a 30-year-old female, the probability of dying within one year from any cause is estimated to be 0.00081,[14] which is lower than the median Pr(death|exposure) from the animal exposure. Thus, based on both Pr(rabid|exposure) and Pr(death|exposure), PEP would be recommended.
Outcome. The dog died one day after the bite. The owner initially refused to submit the dog for testing, but eventually relented. There was a delay of about five days after the bite before testing results were anticipated. The recommendation was to wait for testing results, but the victim decided to start PEP out of an abundance of caution. The dog tested negative for RABV.

© 2023 Charniga K et al. *JAMA Network Open.*

Case study 4: Pennsylvania mountain lion
Scenario. A person in Pennsylvania was feeding a mountain lion [provoked] when he was bitten. The animal was reported to appear healthy.
Estimates. A mountain lion is classified as a large carnivore in the risk assessment tool, and it is a pooled, national estimate. The Pr(rabid|exposure) was 0.0013 (95% CrI: 0.0006 – 0.0021), and the Pr(death|exposure) was 0.0003 (95% CrI: 0.0001 – 0.0006).
Tool recommendation. The Pr(rabid|exposure) is above the risk threshold of 0.0004 (95% CI: 0.0002 – 0.0006). Assuming the victim was a 30-year-old male, the probability of dying within one year from any cause is estimated to be 0.0018,[14] which is higher than the Pr(death|exposure) from the animal exposure. Thus, PEP would be recommended based on Pr(rabid|exposure) but not based on Pr(death|exposure).
Outcome. PEP was recommended, but the victim did not comply.

Case study 5: Pennsylvania cow
Scenario. The wife of a farmer in Pennsylvania reported having saliva contact on her bare arm and hands while feeding a cow. Twenty-one days later, the cow developed symptoms of rabies and later tested positive for RABV.
Estimates. The probability of death given lick to intact skin is 0. Because the saliva did not contact the woman's broken skin or mucous membranes, an exposure did not occur.
Tool recommendation. PEP is not recommended.
Outcome. PEP was not recommended.

Case study 6: Oklahoma skunk
Scenario. A pet skunk bit a veterinarian while being spayed [provoked]. The skunk was purchased from a breeder at eight weeks of age. When the bite occurred, the skunk was five months old, apparently healthy, and unvaccinated. While the skunk had access to the outdoors, the owner reported that the skunk's enclosure provided protection from wildlife.
Estimates. The Pr(rabid|exposure) was 0.13 (95% CrI: 0.11 – 0.15), and the Pr(death|exposure) was 0.03 (95% CrI: 0.02 – 0.05).
Tool recommendation. The Pr(rabid|exposure) is much higher than the risk threshold of 0.0004 (95% CI: 0.0002 – 0.0006). Assuming the veterinarian was a 40-year-old female, the probability of dying from any cause within one year is estimated to be 0.0014,[14] which is lower than the Pr(death|exposure) from the animal exposure. Based on both Pr(rabid|exposure) and Pr(death|exposure), PEP would be recommended. However, the tool does not consider the fact that this animal was kept as a pet and did not have opportunities to interact with rabies reservoir species.
Outcome. Ultimately, the skunk was not tested for RABV, and PEP was not recommended. This case study demonstrates why the tool does not replace expert rabies knowledge and experience.

Case study 7: Georgia cat
Scenario. A known neighborhood cat entered a family's home through an open door. A young child was excited and attempted to grab [provoked] the normal-acting cat, which then scratched and bit the child. An older child was also bitten while trying to get the cat to leave. A neighbor claimed ownership of the cat and assured the family that the cat was up to date on its rabies vaccination; however, the cat nearly always lives outdoors.
Estimates. The Pr(rabid|exposure) was 0.00013 (95% CrI: 0.00011 – 0.00016), while the Pr(death|exposure) was 0.000033 (95% CrI: 0.000019 – 0.000049).
Tool recommendation. The Pr(rabid|exposure) is just below the risk threshold of 0.0004 (95% CI: 0.0002 – 0.0006). Assuming the young child was a 5-year-old male, the probability of dying from any cause within one year is estimated to be 0.00014,[14] which is very similar to the probability of dying from the animal exposure. PEP would not be recommended based on both Pr(rabid|exposure) and Pr(death|exposure).
Outcome. The rabies consult recommended an observation period for the cat of 10 days, avoiding the need to kill and test the cat. The cat did not show symptoms after 10 days, and PEP was not pursued.

© 2023 Charniga K et al. *JAMA Network Open.*

eFigure 8. Risk Thresholds (Vertical Colored/Dashed Lines) Associated With Each Value of the Decision Boundary, P, Considered in 10-Fold Cross-Validation
The y-axis shows the probability that PEP is recommended. The outcome is exclusively binary (0 or 1), but we jittered the points to avoid overlap and improve readability. This plot was made using the full survey dataset (none of the folds from the cross-validation were withheld).

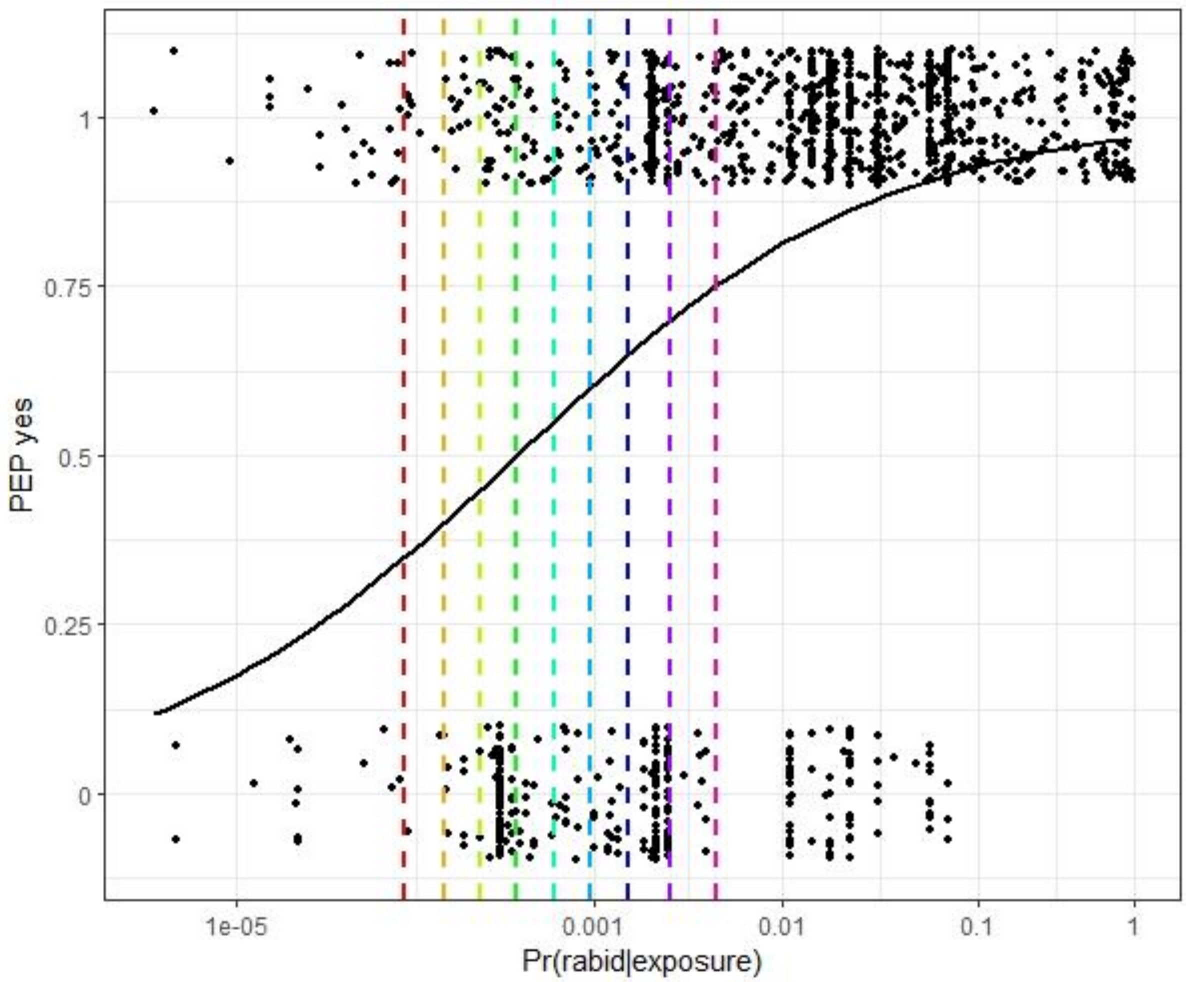

© 2023 Charniga K et al. *JAMA Network Open.*

eTable 2. Evaluation of the Decision Boundary for Logistic Regression Using 10-Fold Cross-Validation

We chose the decision boundary with the highest F-score (bold). F score was calculated as (2 * true positives) / (2 * true positives + false positives + false negatives), using the sums of each of these quantities across the 10 folds.

**P (decision boundary)**	**F-score**	**Risk threshold associated with p using full dataset (all folds)**
P = 0.35	0.854	0.00009
P = 0.40	0.853	0.00015
P = 0.45	0.849	0.00023
**P = 0.50**	**0.862**	**0.00037**
P = 0.55	0.857	0.00059
P = 0.60	0.853	0.0009
P = 0.65	0.845	0.0015
P = 0.70	0.814	0.0026
P = 0.75	0.802	0.0047

Risk threshold corresponds to Pr(rabid|exposure)

© 2023 Charniga K et al. *JAMA Network Open.*

eFigure 9. Violin Plots of the Estimates From the Probability Model, US
There were 1,728 observations for Pr(rabid|exposure) and 41,472 observations for Pr(death|exposure). National estimates and pooled estimates according to the presence of terrestrial RABV variants were only included once (rather than repeated for each jurisdiction) and estimates from lick to intact skin (0 risk of rabies) were removed. Data sources for the estimates included the national rabies surveillance system (2011 – 2020) and the literature.

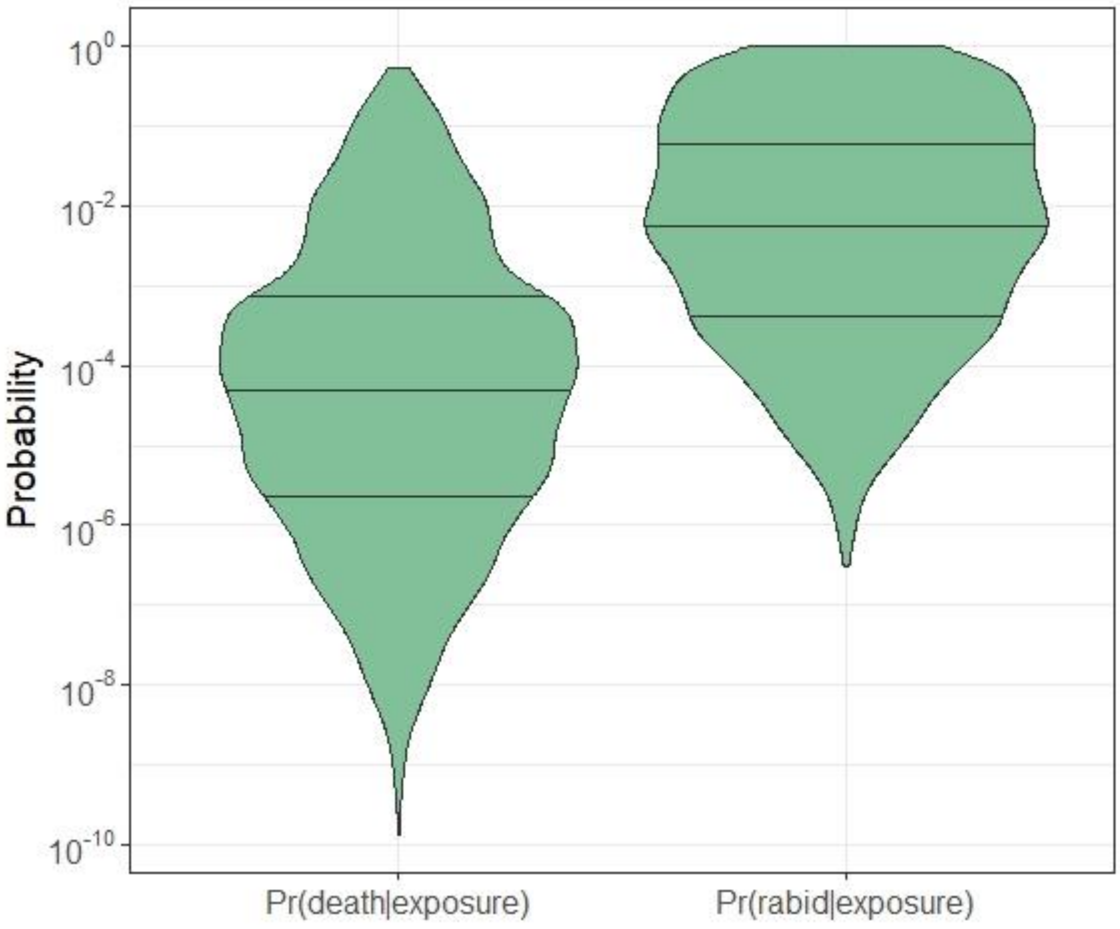

© 2023 Charniga K et al. *JAMA Network Open.*

eResults

Positivity rates. Overall positivity rates for rabies surveillance data collected between 2011 – 2020 are shown in **eTable 1**. Positivity rates were highest for mongooses (0.83, 95% confidence interval [CI]: 0.78 – 0.87) and skunks (0.29, 95% CI: 0.29 – 0.30). The lowest positivity rate was among pocket pets (0, 95% CI: 0 – 0.017). **eFigure 2** shows the positivity rates for RABV across animal categories for each year from 2011 – 2020. The positivity rates for most animals are consistent across this period, except for arctic fox and mongoose; however, these animals had an average of only 38 and 28 samples each year, respectively. Positivity rates for bats, cats, dogs, raccoons, skunks, and foxes are shown by U.S. jurisdiction in **eFigures 3 – 4**. **eFigure 5** shows the positivity rates for cattle, equine, hoofed animals, rodents of unusual size, and sheep and goats. The positivity rates were significantly lower for cattle, equine, and sheep and goats in jurisdictions without terrestrial RABV compared to those with terrestrial RABV. **eFigure 6** shows positivity rates across bat species. The overall positivity rate for bats was 0.060 (95% CI: 0.061 – 0.062). Among bat species, the highest positivity was observed among the Canyon bat (*Parastrellus hesperus*, 0.49, 95% CI: 0.42 – 0.57).

Parameter estimates for health status. Overall, we obtained data on 3,122 samples in which health status and RABV test result were reported. Including the USDA data, 16 jurisdictions were represented, covering 17 out of 23 animal categories across 6 years. Most of the samples (84%) were raccoons. We found that animals that were ill or acting strangely were 20.2 times as likely to test positive for RABV compared to those that were apparently healthy (risk ratio = % rabies among sick animals / % rabies among healthy animals = [39/(39+721)] / [6/(6+2356)] = 20.2). We also obtained the following conditional probabilities, where sick represents animals that were ill or acting strangely:

$$P(sick|rabid) = \frac{39}{45} = 0.87$$

$$P(sick|not\ rabid) = \frac{721}{3077} = 0.23$$

$$P(healthy|rabid) = \frac{6}{45} = 0.13$$

$$P(healthy|not\ rabid) = \frac{2356}{3077} = 0.77$$

Our risk ratio for health status is similar to risk ratios reported by Medley et al. for specific clinical factors associated with rabid dogs in Haiti. They found risk ratios of 31.2 (95% CI: 20.0 – 48.7), 19.7 (95% CI: 11.7 – 33.0), and 15.9 (95% CI: 7.5 – 33.8) for hypersalivation, paralysis, and lethargy, respectively.[19]

Parameter estimates for exposure circumstances. Out of 496 samples, fourteen animal categories were represented in the surveillance data from Arizona that were used to estimate the increased risk of rabies given exposure circumstances; 44% of samples were either dogs or cats, while 31% of samples were bats. Animals that were involved in an unprovoked exposure were 2.8 times as likely to test positive for RABV compared to animals involved in provoked exposures (risk ratio = % rabies among unprovoked exposure / % rabies among provoked exposure = [27/(27+111)] / [25/(25+333)] = 2.8). The data were too sparse to stratify by domestic (cat, cow, dog, goat, horse, pig, and sheep) vs wild animal; however, the risk ratio for all domestic animals was 14.5 compared to 1.6 for wild animals. We obtained the following conditional probabilities from the 2x2 table:

$$P(unprovoked|rabid) = \frac{27}{52} = 0.52$$

$$P(unprovoked|not\ rabid) = \frac{111}{444} = 0.25$$

$$P(provoked|rabid) = \frac{25}{52} = 0.48$$

$$P(provoked|not\ rabid) = \frac{333}{444} = 0.75$$

© 2023 Charniga K et al. *JAMA Network Open.*

Parameter estimates for anatomical location and severity of exposure. We found four papers published between 1912 and 2010 that met our inclusion criteria. In general, the data were scarce and of low quality (**eTable 3**). A range for the probabilities or a point estimate was often reported. Sample sizes were rarely reported. Only two studies reported primary data, which were from Iran and Tanzania. Babes reported the most estimates. He summarized historical data for which the geographical location was ambiguous.[20] Vaidya et al. estimated probabilities of death for various exposures using expert opinion.[21] The studies differed with respect to the rabies status of the animals: Shim et al. reported probabilities for suspect rabid animals,[22] while Babes and Baltazard et al. reported some probabilities for animals that were proven to be rabid, often by the death of one or more of the victims.[20,23] Notably, diagnosis of RABV was not possible until 1903.[24] We categorized the estimates into four anatomical locations: head/neck, upper extremities, torso, and lower extremities. We noted in **eTable 3** when a more specific location (e.g., hands or face) was reported.

The geographical location of the exposure is not relevant for these parameter estimates because the animals were either rabid or strongly suspected to be rabid. However, the species of animal is important because it is related to severity of exposure. Most exposures in our review were from rabid cats, dogs, or wolves and may not generalize well to rabies reservoir species in the United States; for example, multiple bites from a wolf would be more severe than multiple bites from a North American bat due to larger body size and teeth and hence, more inoculated virus.

Distributions for anatomical location and severity of exposure. We used distributions to summarize the data from **eTable 3** to obtain **eTable 4**. We categorized the estimates into seven levels of severity: multiple bites, single bite, scratch or superficial bite (with teeth), scratch (with nails/claws), mucosal, lick to broken skin, and lick to intact skin. Ten out of 19 combinations of location and severity obtained from the literature were point estimates (**eTable 3**). We used a binomial distribution for one combination because the sample size was known. We used triangular distributions for the remaining combinations, following Cleaveland et al. (**eTable 4**).[25] A triangular distribution is a continuous probability distribution with lower limit a, upper limit b and mode c, where $a < b$ and $a \leq c \leq b$. This distribution is often used when data are scarce. We identified the minimum and maximum values for the triangular distribution from the available data for each combination of location and severity. We used the midpoint of the minimum and maximum for the mode, c. For parameter estimates that had Shim et al.[22] as the only reference, we used the point estimate for the mode and the 95% confidence intervals as a and b. Alternatively, we could have used uniform distributions instead of triangular distributions.

eFigure 10 shows the probability distributions for single and multiple bite exposures. We only had one reference informing the distribution of single bite to the lower extremities. Based on the data for multiple bite exposures, we adjusted the parameter estimate for single bite to the lower extremities by subtracting 0.12 each from the minimum, mode, and maximum, so that the minimum and mode would be 0.

We used fold changes to estimate parameters for combinations of location and severity that were missing from the literature. We used the mode values for multiple bites and single bite to calculate fold changes (increased risk) for torso to upper extremities and for upper extremities to head/neck. The fold changes were used to impute missing values for other combinations of location and severity. Four combinations were missing: scratch or superficial bite (teeth) for both torso and upper extremities and scratch (nails/claws) for both head/neck and torso. The average fold change was 2.3 and 1.9 for torso to upper extremities and for upper extremities to head/neck, respectively, across multiple bites and single bite exposures. As we did not have enough information to calculate a fold change for lower extremities to torso, we used the same value for lower extremities as torso for scratch or superficial bite (teeth).

In addition to the missing combinations noted above, three parameter estimates for severity were not specific to a location: mucosal, lick to broken skin, and lick to intact skin. In each case, we applied the same non-specific estimate to all four location categories. The total number of location and severity combinations was 28 (4 locations times 7 levels of severity).

Risk model (updating probabilities). We used the following equations to update the probabilities in our model:

$$P(A|B) = \frac{P(B|A)\,P(A)}{P(B)} \tag{1}$$

© 2023 Charniga K et al. *JAMA Network Open.*

$$P(A|B) = \frac{P(B|A)\,P(A)}{P(B|A)\,P(A) + P(B|A^C)\,P(A^C)} \quad (2)$$

where Equation (1) is Bayes' rule, and Equation (2) is derived using the law of total probability.

The distributions of all the estimates from the probability model are shown in **eFigure 9**. We obtained 1,728 unique observations in the resulting dataset for Pr(rabid|exposure). The number of observations was determined in the following way: 2 jurisdiction-specific species can be vaccinated, so 2 species * 51 jurisdictions * 8 combinations of health status, exposure circumstances, and vaccination status = 816. The remaining 4 jurisdiction-specific species cannot be vaccinated, so 4 species * 51 jurisdictions * 4 combinations of health status and exposure circumstances. We subtracted 5 species * 4 combinations of health status and exposure circumstances because from 2011 – 2020, no samples of raccoons, skunks, or foxes were available for Puerto Rico, and no samples of raccoons or foxes were available for Alaska, which equals 816 – 20 = 796. Of 22 national and pooled estimates according to the presence of terrestrial RABV variants, 7 can be vaccinated (equine, sheep and goats, and cattle are counted twice here for jurisdictions with/without terrestrial RABV), so 7 species * 8 combinations of health status, exposure circumstances, and vaccination status = 56. The remaining 15 species (rodents of unusual size and hoofed animals are counted twice for jurisdictions with/without terrestrial RABV) cannot be vaccinated, so 15 species * 4 combinations of health status and exposure circumstances = 60. The sum is 1,728. There were 5,496 total observations after national and pooled estimates according to the presence of terrestrial RABV variants were repeated for each jurisdiction.

Similarly, we obtained 41,472 unique estimates for Pr(death|exposure). The number of observations was determined as 1,728 * 28 combinations of the probability of death from rabies according to anatomical location and severity of exposure = 48,384. Then we excluded estimates in which the exposure involved lick to intact skin (rabies risk of 0) = 48,384 – (1,728 * 4 anatomical locations * 1 level of severity) = 41,472. There were 153,888 total estimates for Pr(death|exposure) after national and pooled estimates according to the presence of terrestrial RABV variants were repeated for each jurisdiction and estimates for exposures involving licks to intact skin (0 rabies risk) were included.

Survey. Fifty surveys were completed from 31 jurisdictions (**eFigure 11**). Jurisdictions returned between 0 and 4 surveys. The spatial distribution of respondents covered all major RABV variant regions in the United States.[4] Respondents had an average of 9.2 years of experience making professional recommendations about rabies PEP (range 0 – 30). Forty-nine respondents worked at the state public health level, while one worked at the local level. Respondents reported a range of non-mutually exclusive education and experience, including clinical (N = 9), epidemiology (N = 40), veterinary medicine (N = 31), wildlife ecology or zoology (N = 2), health education (N = 1), and public health (N = 1). The number of times PEP was recommended out of 24 scenarios (sum of "agree" and "strongly agree") was 18.2 on average (range 11 – 24). There was a non-linear (triangular) correlation between years of experience and both the number of times PEP was recommended and risk profile (**eFigure 12**). This relationship suggests that there is greater variability in PEP recommendations among public health officials with less experience compared to those with more experience. Risk profile was calculated by summing: Strongly agree = 1, Agree = 2, Disagree = 3, Strongly disagree = 4. The higher the number, "riskier" the respondent is.

Determining the decision boundary for p in logistic regression. **eTable 2** shows the performance of the logistic regression model from 10-fold cross-validation for each value of the decision boundary, p, considered. We selected p = 0.5 for having the highest F-score. This decision boundary was associated with a risk threshold of approximately 0.0004 in the full dataset (including all the folds).

eFigure 8 shows the risk thresholds associated with each p. There are several false positives using our decision boundary of 0.5. These points are in the lower right corner of **eFigure 8**. False positives are not as concerning as false negatives because it means our recommendations are conservative compared to those of the survey respondents. In other words, the tool would recommend PEP in situations when the subject matter experts do not recommend PEP. False positives may also reflect biases in the surveillance data, which we do not necessarily want to correct for at the expense of incorrectly classifying high-risk exposures as PEP = no. Based on rabies risk assessment guidelines, we expect the surveillance data are over-representative of high-risk exposures. They could also reflect experts making recommendations that are inconsistent with data.

© 2023 Charniga K et al. *JAMA Network Open.*

Model diagnostics for logistic regression. We obtained a Pseudo-R^2 (McFadden) of 0.16, suggesting moderate fit. Pseudo R^2 ranges between 0 and 1; however, values near 1 are exceedingly rare for real data. Values between 0.2 and 0.4 are considered a very good fit.[26]

We checked the four main assumptions of logistic regression, (1) the outcome is binary, (2) the relationship between the logit of the outcome and each predictor variable is linear, (3) there are no extreme outliers in the predictors, and (4) there is no multicollinearity among predictor variables. (4) is not relevant, as we only have one predictor. The outcome is binary, as we collapsed the survey responses into PEP yes vs no. **eFigure 13** shows the plot for (2), demonstrating that the assumption of linearity is met. We identified 73 potentially influential observations in our data that had Cook's distance > 4/N, where N is the total number of data points, 1,194 (**eFigure 13**); however, all standardized residuals had an absolute value < 3. Among the 73 potential outliers, there were 30 instances where PEP was recommended, yet Pr(rabid|exposure) was low, and there were 43 instances where PEP was not recommended but Pr(rabid|exposure) was high (0.02 >). Omitting these observations, we obtained a risk threshold of 0.0008.

Sensitivity analyses on survey data. When we only included respondents with at least 9 years of experience, we found a similar risk threshold compared to the main analysis: 0.0003.

Using only survey responses consisting of "strongly agree" and "strongly disagree," we obtained a risk threshold of 0.0004 which was the same as that obtained in the main analysis.

Outliers in survey data. Out of 1,194 non-missing observations (survey scenario matched with underlying Pr(rabid|exposure) estimate), 194 (16%) observations had a median above the risk threshold, yet PEP was not recommended. Of these, 167 were "disagree" and only 27 were "strongly disagree." For "strongly disagree" (**eTable 5**), 2 out of 27 observations involved animal categories for which a national estimate was used. Thirteen jurisdictions were represented among the observations where the subject matter experts' opinion from the survey did not match the model-based PEP recommendation. Arizona had the highest number of observations in the group with five. The highest Pr(rabid|exposure) for which PEP was not recommended was associated with a rodent of unusual size in Tennessee (0.093, 95% credible interval [CrI]: 0.084 – 0.100).

© 2023 Charniga K et al. *JAMA Network Open.*

eFigure 10. Probability Distributions for Developing Rabies From Single and Multiple Bite Exposures

Colors indicate anatomical location. (A) Multiple bites, (B) unadjusted single bite, using all available data from the historical literature, and (C) adjusted single bite. The probability of developing rabies was decreased for single bite to the lower extremities in (C) to reflect a higher risk from multiple bites.

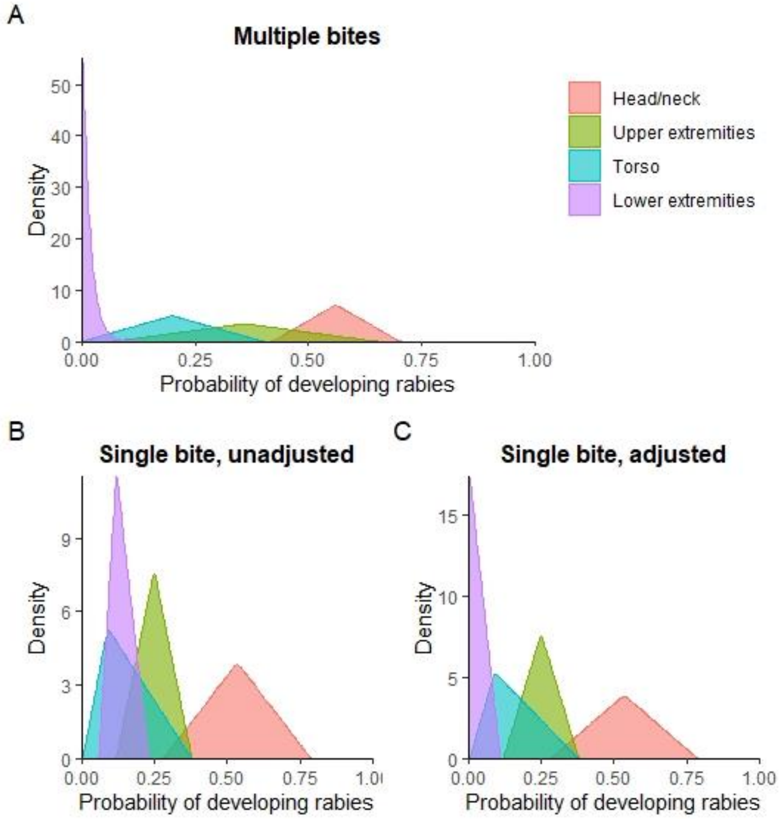

© 2023 Charniga K et al. *JAMA Network Open.*

eTable 3. Estimates of the Probability of Developing Rabies According to Anatomical Location and Severity of Exposure From the Literature

Location category	Specific location in reference	Severity	Description	Method[a]	Sample size	Value (95% CI)[b]	Ref
Head/neck		Multiple bites	Probability of dying from rabies after multiple severe bites to the eye area, nose area, or other parts of the head by rabid dogs or cats.	Estimated from historical data on people who did not receive PEP.		Range: 0.50-0.70	[20]
Head/neck		Almost all bites of exceptional severity	Probability of dying from rabies after bite(s) to the head by a wolf that was proven to be rabid by the deaths of one or more of its victims.	Estimated from 13 years of data on wolf bite victims in Iran. Patients were all treated sub-optimally with nerve tissue vaccines (Pereira de Silva type and Semple type).		0.42	[23]
Head/neck		Not reported but likely severe as the rabid animal was a wolf	Probability of dying of rabies after bite(s) to the head by a wolf that was proven to be rabid by the deaths of one or more of its victims.	Estimated during an outbreak of rabies in southern Iran among victims who either did not receive PEP.		0.545	[23] citing Gremliza 1953[c]
Head/neck		Not reported but likely severe as the rabid animal was a wolf	Probability of dying of rabies after bite(s) to the head by a wolf that was proven to be rabid by the deaths of one or more of its victims.	Estimated based on historical case series from several countries published before the discovery of PEP.		0.45	[23] citing Nicolic 1952[c]
Upper extremities		Multiple bites	Probability of dying of rabies after multiple severe bites to the upper extremities (uncovered by clothes) by rabid wolves.	Estimated from historical data on people who did not receive PEP.		0.40	[20]

© 2023 Charniga K et al. *JAMA Network Open.*

Upper extremities		Almost all bites of exceptional severity	Probability of dying from rabies after bite(s) to the upper limbs by a wolf that was proven to be rabid by the deaths of one or more of its victims.	Estimated from 13 years of data on wolf bite victims in Iran. Patients were all treated sub-optimally with nerve tissue vaccines (Pereira de Silva type and Semple type).		0.13	[23]
Upper extremities		Not reported but likely severe as the rabid animal was a wolf	Probability of dying of rabies after bite(s) to the arms by a wolf that was proven to be rabid by the deaths of one or more of its victims.	Estimated during an outbreak of rabies in southern Iran among victims who did not receive PEP.	10	0.30 (0.07-0.65)	[23] citing Gremliza 1953[c]
Torso		Multiple bites	Probability of dying of rabies after multiple severe bites with the removal of soft parts from the trunk by rabid wolves.	Estimated from historical data on people who did not receive PEP.		0.40	[20]
Torso		Almost all bites of exceptional severity	Probability of dying from rabies after bite(s) to the trunk by a suspect rabid wolf.	Estimated from 13 years of data on wolf bite victims in Iran. Patients were all treated sub-optimally with nerve tissue vaccines (Pereira de Silva type and Semple type).	60	0 (0-0.06)	[23]
Lower extremities		Almost all bites of exceptional severity	Probability of dying from rabies after bite(s) to the lower limbs by a suspect rabid wolf.	Estimated from 13 years of data on wolf bite victims in Iran. Patients were all treated sub-optimally with nerve tissue vaccines (Pereira de Silva type and Semple type).	5	0 (0-0.52)	[23]
Head/neck		Single bite	Probability of dying of rabies after a bite to head/neck by a suspect rabid animal.	Estimated from contact-tracing data collected on the outcome of untreated bite injuries produced by rabid animals in Tanzania.		0.55 (0.28–0.79)	[22]

© 2023 Charniga K et al. *JAMA Network Open.*

Head/neck	Face	Single bite	Probability of dying of rabies after single bite to the face, not too deep, from a rabid dog, cat, or wolf.	Estimated from historical data on people who did not receive PEP.		Range: 0.30-0.40	[20]
Upper extremities		Single bite	Probability of dying of rabies after a bite to the upper extremities by a suspect rabid animal.	Estimated from contact-tracing data collected on the outcome of untreated bite injuries produced by rabid animals in Tanzania.		0.22 (0.12–0.38)	[22]
Upper extremities	Hands	Single bite	Probability of dying of rabies after a deep bite to the hands, especially the fingertips, by rabid cats, dogs, or wolves.	Estimated from historical data on people who did not receive PEP.		0.15-0.20	[20]
Torso		Single bite	Probability of dying of rabies after a bite to the torso by a suspect rabid animal.	Estimated from contact-tracing data collected on the outcome of untreated bite injuries produced by rabid animals in Tanzania.		0.09 (0.005–0.38)	[22]
Lower extremities		Single bite	Probability of dying of rabies after a bite to the lower extremities by a suspect rabid animal.	Estimated from contact-tracing data collected on the outcome of untreated bite injuries produced by rabid animals in Tanzania.		0.12 (0.06–0.23)	[22]
Head/neck	Face	Scratch or superficial bite (teeth)	Probability of dying of rabies after a scratch or superficial bite from a rabid animal's (cats, dogs, or wolves) teeth.	Estimated from historical data on people who did not receive PEP.		0.10	[20]
Upper extremities	Hands	Scratch or superficial bite (teeth)	Probability of dying of rabies after a scratch or superficial bite from a rabid animal's (cats, dogs, or wolves) teeth.	Estimated from historical data on people who did not receive PEP.		0.05	[20]
Upper/lower extremities	Extremities	Scratch (nails/claws)	Probability of dying of rabies after a scratch from a rabid animal's nails or claws.	Estimated from historical data on people who did not receive PEP.		0.001	[20]

© 2023 Charniga K et al. *JAMA Network Open.*

		Mucosal	Probability of dying of rabies after mucous membrane (i.e., mouth, nasal cavity) contact with saliva from a rabid animal.	Estimated from historical data on people who did not receive PEP.		0.001	[20]
		Lick to broken skin	Probability of dying of rabies after a lick from a rabid cat or dog over skin with recent superficial scratches (area not washed or cleaned).	Delphi method was used to obtain expert group consensus.		Range: 0.000001 - 0.0001	[21]
		Lick to broken skin	Probability of dying of rabies after contact with the saliva of a rabid animal on recent cuts and scratches.	Estimated from historical data on people who did not receive PEP.		0.001	[20]
		Lick to intact skin	Probability of dying of rabies after contact with the saliva of a rabid animal on closed scratches older than 24 hours.	Estimated from historical data on people who did not receive PEP.		0	[20]

[a]PEP: post-exposure prophylaxis
[b]95% exact binomial confidence intervals were calculated when not provided in the original references
[c]Nicolic and Gremliza references are not cited directly as they are in German

© 2023 Charniga K et al. *JAMA Network Open.*

eTable 4. Final Parameter Estimates for Anatomical Location and Severity of Exposure

Location category	Severity	Distribution	Value	Ref
Head/neck	Multiple bites	Triangular	Minimum = 0.42, mode = 0.56, maximum = 0.70	[20,23] (also Gremliza 1953 and Nicolic 1952)[a]
Upper extremities	Multiple bites	Triangular	Minimum = 0.07, mode = 0.36, maximum = 0.65	[20,23] (also Gremliza 1953)[a]
Torso	Multiple bites	Triangular	Minimum = 0, mode = 0.20, maximum = 0.40	[20,23]
Lower extremities	Multiple bites	Binomial	*p* = 0, *n* = 60	[23]
Head/neck	Single bite	Triangular	Minimum = 0.28, mode = 0.535, maximum = 0.79	[20,22]
Upper extremities	Single bite	Triangular	Minimum = 0.12, mode = 0.25, maximum = 0.38	[20,22]
Torso	Single bite	Triangular	Minimum = 0.005, mode = 0.09, maximum = 0.38	[22]
Lower extremities	Single bite	Triangular[b]	Minimum = 0, mode = 0, maximum = 0.11	[22]
Head/neck	Scratch or superficial bite (teeth)	Point estimate	0.10	[20]
Upper extremities	Scratch or superficial bite (teeth)	Point estimate	0.05	[20]
Torso	Scratch or superficial bite (teeth)	Point estimate	0.02	Estimated from fold change
Lower extremities	Scratch or superficial bite (teeth)	Point estimate	0.02	Estimated from fold change
Head/neck	Scratch (nails/claws)	Point estimate	0.002	Estimated from fold change
Upper extremities	Scratch (nails/claws)	Point estimate	0.001	[20]
Torso	Scratch (nails/claws)	Point estimate	0.001	Estimated from fold change
Lower extremities	Scratch (nails/claws)	Point estimate	0.001	[20]
Not specific	Mucosal	Point estimate	0.001	[20]

© 2023 Charniga K et al. *JAMA Network Open.*

Not specific	Lick to broken skin	Triangular	Minimum = 0.000001,<br>mode = 0.0005,<br>maximum = 0.001	[20,21]
Not specific	Lick to intact skin	Point estimate	0	[20]

[a]Nicolic and Gremliza references are not cited directly as they are in German

[b]We adjusted this distribution by subtracting 0.12 from the minimum, mode, and maximum values

© 2023 Charniga K et al. *JAMA Network Open.*

eFigure 11. Spatial Distribution of Completed Surveys
50 surveys were returned from 31 jurisdictions in the United States, including one from Puerto Rico, which is not shown on the map. South Carolina returned the most surveys (N = 4).

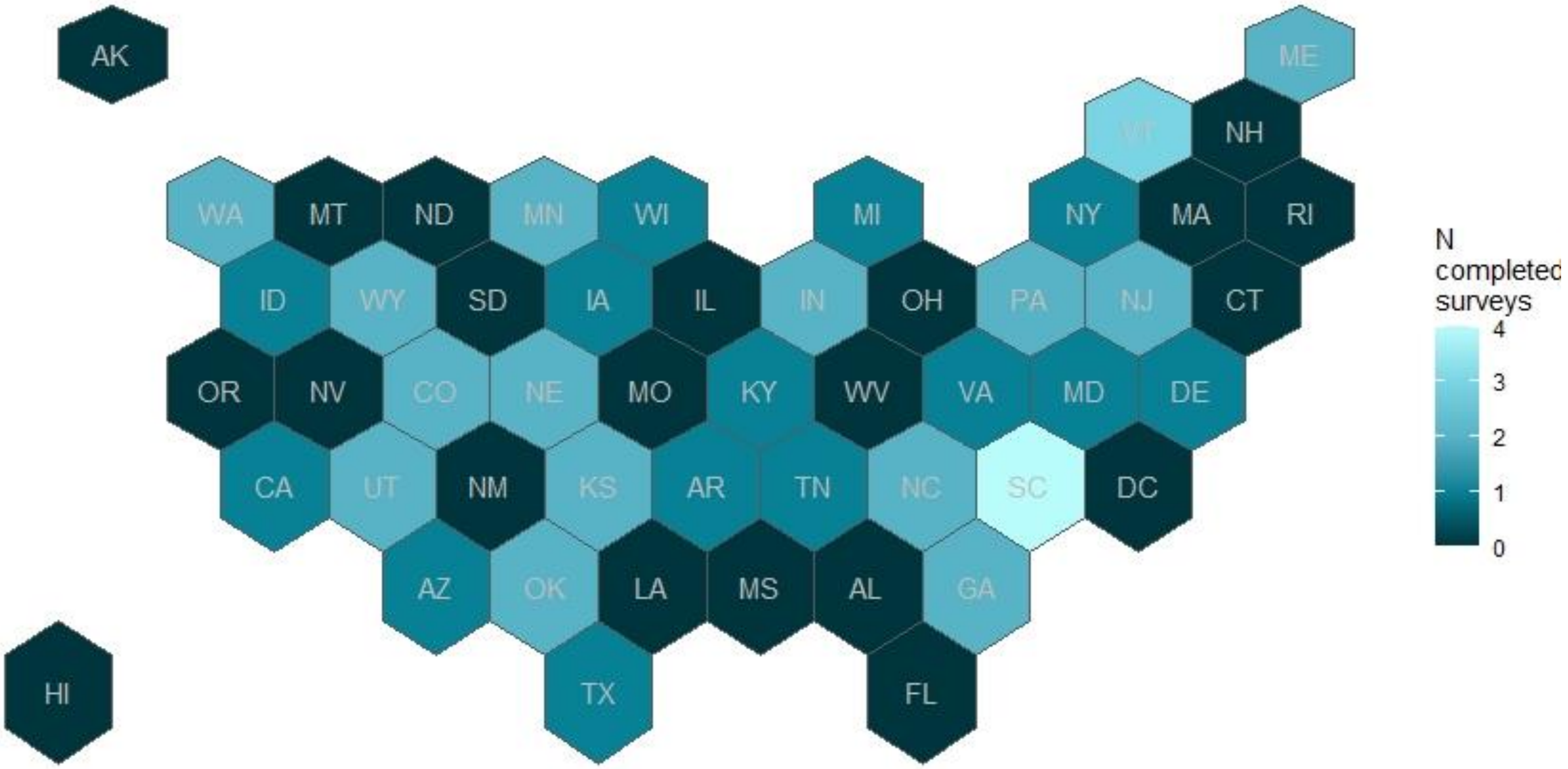

© 2023 Charniga K et al. *JAMA Network Open.*

eFigure 12. Risk Profile of Survey Respondents According to Years of Experience
A higher risk score indicates post-exposure prophylaxis (PEP) was recommended less frequently, while lower risk scores indicate more conservative PEP recommendations.

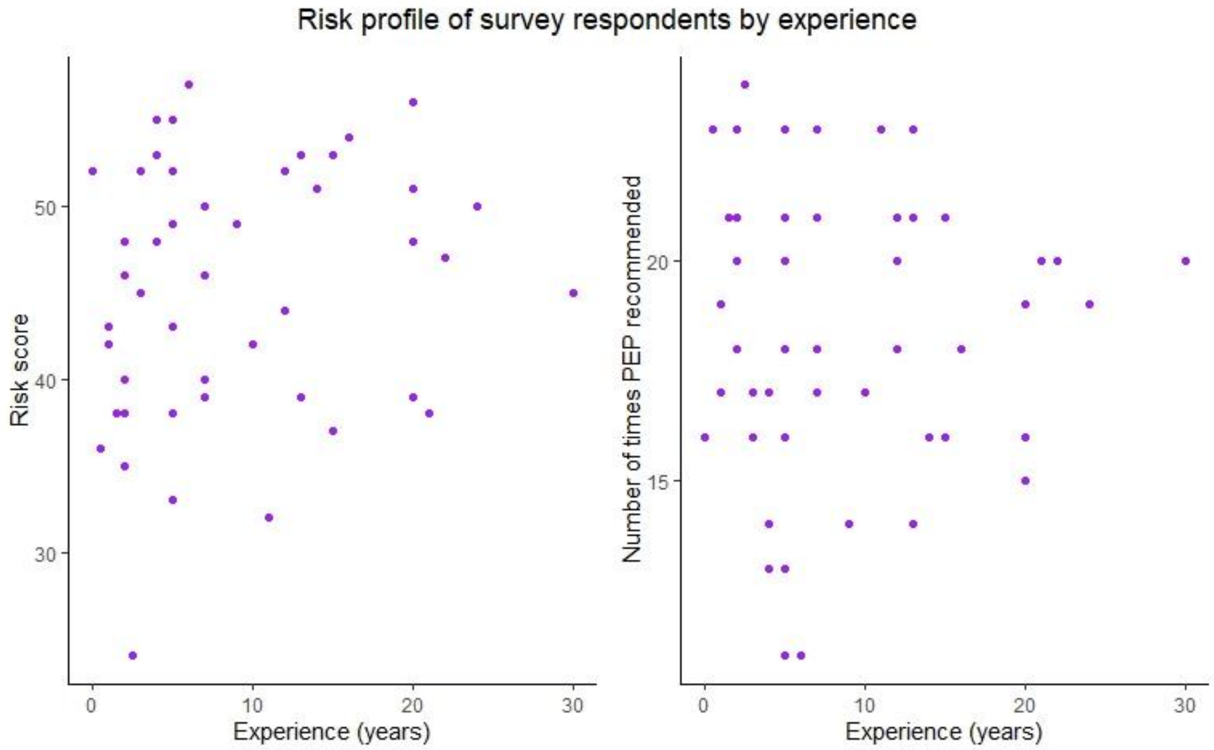

© 2023 Charniga K et al. *JAMA Network Open.*

eTable 5. Survey Scenarios Above the Estimated Threshold for Which Postexposure Prophylaxis Was Not Recommended ("Strongly Disagree," N = 27)

Jurisdiction	Animal	Provoked	Apparently healthy	Vaccinated	Pr(rabid|exposure) (95% CrI[d])
TN	Rodents of unusual size	Yes	No	NA	0.093 (0.084 – 0.100)
AZ	Fox	Yes	Yes	NA	0.062 (0.049 – 0.076)
AZ	Bobcat[a]	Yes	Yes	NA	0.017 (0.016 – 0.019)
CO, NC	Equine	No	Yes	No/unknown	0.012 (0.011 – 0.014)
AZ, CO, NC, OK, TN, VT	Hoofed animals	Yes	Yes	NA	0.0026 (0.0020 – 0.0033)
PR	Cat	No	Yes	Yes	0.0022 (0.0013 – 0.0034)
AZ, CA, KS, NC, OK, PR, VT[b]	Sheep and goats	Yes	Yes	No/unknown	0.0022 (0.0018 – 0.0026)
AZ	Mesocarnivores[a]	Yes	Yes	NA	0.0021 (0.0018 – 0.0024)
IN, WA[c]	Hoofed animals	Yes	Yes	No/unknown	0.0019 (0.00007 – 0.0100)
CO	Dog	No	Yes	No/unknown	0.0010 (0.0005 – 0.0017)
PA	Cat	No	Yes	Yes	0.00047 (0.00042 – 0.00051)
GA	Cat	No	Yes	Yes	0.00041 (0.00035 – 0.00048)
VT	Dog	Yes	No	Yes	0.00041 (0 – 0.00123)

[a]National-level estimates
[b]Two respondents from VT classified the scenario as "strongly disagree"
[c]Jurisdictions without terrestrial RABV variants
[d]CrI: credible intervals

© 2023 Charniga K et al. *JAMA Network Open.*

eFigure 13. Logistic Regression Diagnostics
(A) Checking the linearity assumption. Visually, we can see that the predictor is linearly associated with the outcome on the logit scale. (B) Checking outliers in the data with Cook's distance. The top three outliers are labeled. Seventy-three observations had a Cook's distance above 4/N, where N is the total number of observations; this threshold is often used to flag influential points.

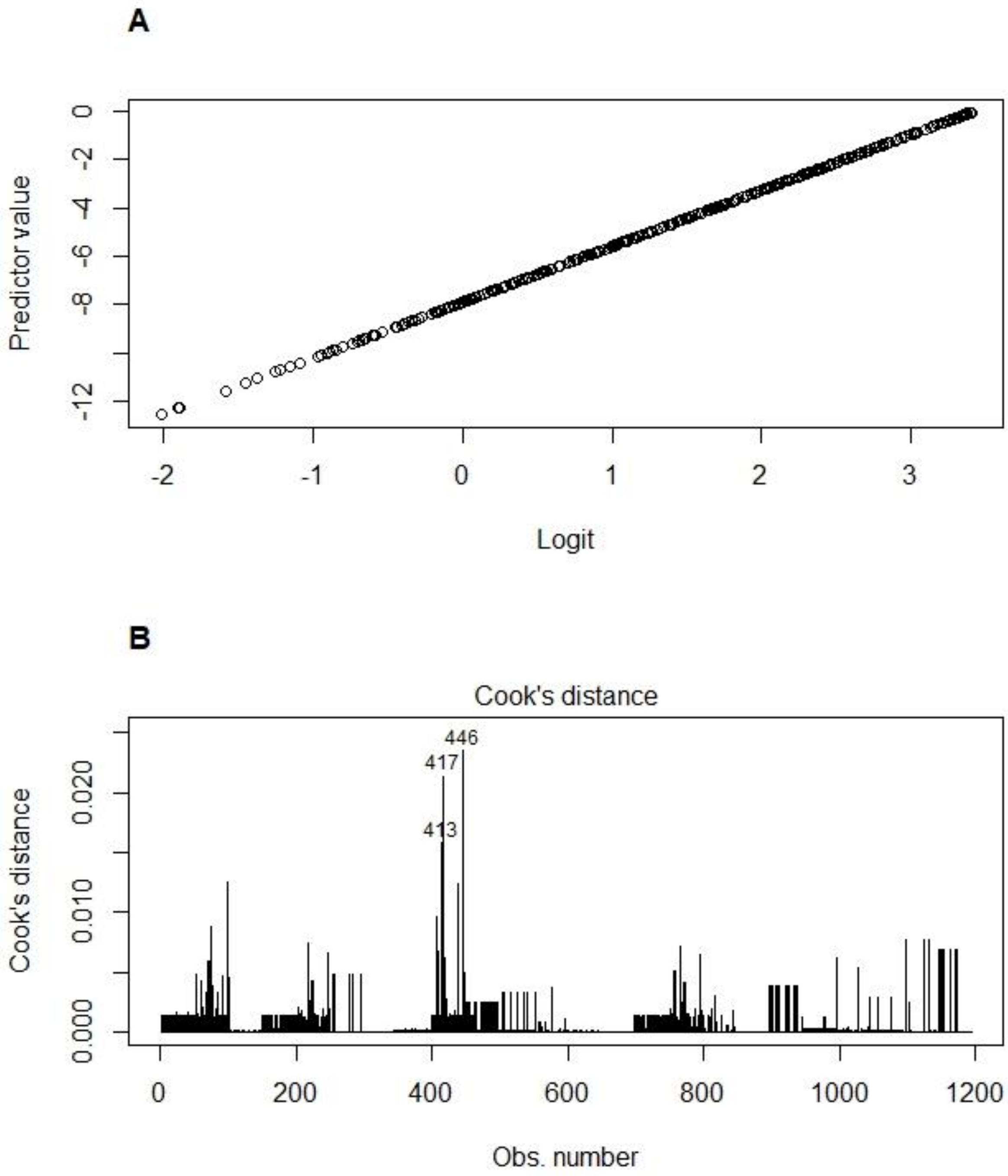

© 2023 Charniga K et al. *JAMA Network Open.*

eReferences

1. National Notifiable Diseases Surveillance System (NNDSS). Rabies, Human. Accessed May 17, 2022, https://ndc.services.cdc.gov/conditions/rabies-human/
2. National Notifiable Diseases Surveillance System (NNDSS). Rabies, Animal. Accessed May 17, 2022, https://ndc.services.cdc.gov/conditions/rabies-animal/
3. U.S. Centers for Disease Control and Prevention. Direct fluorescent antibody test. Accessed May 17, 2022, https://www.cdc.gov/rabies/diagnosis/direct_fluorescent_antibody.html
4. Ma X, Monroe B, Wallace R, et al. Rabies surveillance in the United States during 2019. *JAVMA*. 2021;258(11):1205-1220.
5. U.S. Centers for Disease Control and Prevention. Accuracy of the tests. Updated April 22, 2011. Accessed August 24, 2022, https://www.cdc.gov/rabies/diagnosis/accuracy.html
6. Council of State and Territorial Epidemiologists. Public Health Reporting and National Notification for Animal Rabies. 09-ID-12. Accessed May 17, 2022, https://cdn.ymaws.com/www.cste.org/resource/resmgr/PS/09-ID-12.pdf
7. Schoch C, Ciufo S, Domrachev M, et al. NCBI Taxonomy: a comprehensive update on curation, resources and tools. *Database*. 2020;2020:baaa062.
8. Myers P, Espinosa R, Parr C, Jones T, Hammond G, Dewey T. The Animal Diversity Web. https://animaldiversity.org
9. Florida Department of Health. Rabies Exposure and Risk Assessment. Accessed August 16, 2022, https://www.floridahealth.gov/diseases-and-conditions/rabies/professionals.html
10. Texas Department of State Health Services. Rabies Prevention in Texas. Accessed August 16, 2022, https://dshs.texas.gov/IDCU/disease/rabies/information/prevention/Rabies-Prevention-in-Texas-052722.pdf
11. U.S. Centers for Disease Control and Prevention. Public Health Importance of Rabies. Updated April 6, 2020. Accessed April 11, 2023, https://www.cdc.gov/rabies/location/usa/index.html
12. Etz A. Introduction to the Concept of Likelihood and Its Applications. *Advances in Methods and Practices in Psychological Science*. 2018;1(1):60-69.
13. Forman G, Scholz M. Apples-to-apples in cross-validation studies: pitfalls in classifier performance measurement. *ACM SIGKDD Explorations Newsletter*. 2010;12(1):49-57. doi:https://doi.org/10.1145/1882471.1882479
14. U.S. Social Security Administration. Period Life Table, 2019, as used in the 2022 Trustees Report. Accessed August 18, 2022, https://www.ssa.gov/oact/STATS/table4c6.html
15. National Safety Council. Odds of Dying. Accessed August 18, 2022, https://injuryfacts.nsc.org/all-injuries/preventable-death-overview/odds-of-dying/
16. Messenger S, Smith J, Orciari L, Yager P, Rupprecht C. Emerging Pattern of Rabies Deaths and Increased Viral Infectivity. *Emerg Infect Dis*. 2003;9(2):151-154.
17. Rupprecht C, Briggs D, Brown C, et al. Use of a Reduced (4-Dose) Vaccine Schedule for Postexposure Prophylaxis to Prevent Human Rabies: Recommendations of the Advisory Committee on Immunization Practices. *MMWR*. 2010;59(RR02):1-9.
18. Pieracci E, Pearson C, Wallace R, et al. Vital Signs: Trends in Human Rabies Deaths and Exposures — United States, 1938–2018. *MMWR Morb Mortal Wkly Rep*. 2019;68:524-528.
19. Medley A, Millien M, Blanton J, et al. Retrospective Cohort Study to Assess the Risk of Rabies in Biting Dogs, 2013–2015, Republic of Haiti. *Trop Med Infect Dis*. 2017;2(2):14.
20. Babes V. *Traité de la Rage*. Librairie J.-B. Baillière et Fils; 1912. https://gallica.bnf.fr/ark:/12148/bpt6k5462676f/f13.item.texteImage
21. Vaidya S, Manning S, Dhankhar P, et al. Estimating the risk of rabies transmission to humans in the U.S.: a delphi analysis. *BMC Public Health*. 2010;10:278.

© 2023 Charniga K et al. *JAMA Network Open.*

22. Shim E, Hampson K, Cleaveland S, Galvani A. Evaluating the cost-effectiveness of rabies post-exposure prophylaxis: A case study in Tanzania. *Vaccine*. 2009;27(51):7167-7172.
23. Baltazard M, Ghodssi M. Treatment of persons bitten by rabid wolves in Iran. *Bull Wld Hlth Org*. 1954;10:797-803.
24. Baer G. Jackson A, Wunner W, eds. *Rabies (Second Edition)*. Academic Press; 2007:1-22.
25. Cleaveland S, Fèvre E, Kaare M, Coleman P. Estimating human rabies mortality in the United Republic of Tanzania from dog bite injuries. *Bull Wld Hlth Org*. 2002;80(4):304-310.
26. McFadden D. Quantitative Methods for Analyzing Travel Behaviour of Individuals: Some Recent Developments. *Cowles Foundation Discussion Papers*. 1977;707:35.
27. Brown C, Slavinski S, Ettestad P, Sidwa T, Sorhage F. Compendium of Animal Rabies Prevention and Control, 2016. *JAVMA*. 2016;248(5):505-517.

© 2023 Charniga K et al. *JAMA Network Open.*
