# Supplementary material for: Risk of Rabies and Implications for Postexposure Prophylaxis Administration in the US
Source: JAMA Netw Open. 2023 Jun 9;6(6):e2317121. doi: 10.1001/jamanetworkopen.2023.17121 (PMC10257100; doi:10.1001/jamanetworkopen.2023.17121)
Supplement: Supplement 2. — Data Sharing Statement [file jamanetwopen-e2317121-s002.pdf]

Data Sharing Statement

Charniga. Risk of Rabies and Implications for Postexposure Prophylaxis Administration in the US. *JAMA Netw Open*. Published June 09, 2023. doi:10.1001/jamanetworkopen.2023.17121

Data

Data available: Yes
Data types: Data (not involving human participants)
How to access data: Data and code for the analysis will be available on GitHub following publication (https://github.com/kcharniga/rabies_shiny). The Shiny apps will be available to external partners through CDC's Posit Connect server using the AMD portal without the need to download R/RStudio (login to SAMS is required). Finally, the apps are publicly available at the following links, but each app is limited to 25 active hours of use per month. All animals: https://kellycharniga.shinyapps.io/RabiesRiskTool/?_ga=2.192835374.528424846.1680190191-1149591592.1678282481; bat species: https://kellycharniga.shinyapps.io/RabiesRiskToolBatSpecies/?_ga=2.189712303.528424846.1680190191-1149591592.1678282481.
When available: With publication

Supporting Documents

Document types: Statistical/analytic code
How to access documents: https://github.com/kcharniga/rabies_shiny
When available: With publication

Additional Information

Who can access the data: The data will be publicly available
Types of analyses: The data can be used for any purpose
Mechanisms of data availability: The data will be available on a public GitHub repository
